# RAFT with Light: A User Guide to Using Thiocarbonylthio Compounds in Photopolymerizations

**DOI:** 10.1021/acspolymersau.4c00101

**Published:** 2025-03-27

**Authors:** Magdalena A. Beres, Cyrille Boyer, Matthias Hartlieb, Dominik Konkolewicz, Greg G. Qiao, Brent S. Sumerlin, Sébastien Perrier

**Affiliations:** a Department of Chemistry, 2707 University of Warwick, Coventry CV4 7AL, United Kingdom; b Cluster for Advanced Macromolecular Design, School of Chemical Engineering, The University of New South Wales, Sydney, NSW 2052, Australia; c Institute of Chemistry, 26583 University of Potsdam, Karl-Liebknecht-Str. 24-25, 14476 Potsdam, Germany; d Fraunhofer Institute for Applied Polymer Research (IAP), Geiselbergstraße 69, 14476 Potsdam, Germany; e Department of Chemistry and Biochemistry, 6403 Miami University, 651 E High St, Oxford, Ohio 45056, United States; f Department of Chemical Engineering, The University of Melbourne, Melbourne 3010, Australia; g George and Josephine Butler Polymer Research Laboratory, Center for Macromolecular Science and Engineering, Department of Chemistry, 3463 University of Florida, Gainesville, Florida 32611-7200, United States; h Warwick Medical School, 2707 University of Warwick, Coventry, CV4 7AL, United Kingdom; i Monash Institute of Pharmaceutical Sciences, Monash University, Parkville, Victoria 3052, Australia

**Keywords:** Reversible addition−fragmentation chain-transfer polymerization, photoiniferter, PET-RAFT, photopolymerization, photoRDRP, light, radical polymerization, photomediated polymerization, reversible-deactivation radical polymerization

## Abstract

This perspective offers an in-depth guide to photopolymerizations mediated with thiocarbonylthio compounds, with a particular focus on photoiniferter and photoinduced energy/electron transfer RAFT (PET-RAFT) polymerizations, focusing on practical considerations. It is designed to provide both newcomers and experts with the practical knowledge needed to harness light-mediated polymerizations for innovative applications. The discussion begins with an overview of conventional RAFT polymerization and proceeds to highlight the distinctive advantages of the photomediated processes. The photochemical behavior of thiocarbonylthio compounds, along with the selection of appropriate light wavelengths, is critically examined for its impact on polymerization kinetics and optimization of polymer properties. Key parameters influencing polymerization success—such as catalyst selection, solvent choice, light intensity, and temperature—are explored in detail. The importance of oxygen tolerance and end-group fidelity is also addressed, as these factors are essential for achieving well-defined polymers. Additionally, reactor configurations are reviewed, focusing on the roles of light sources, reactor geometry (batch versus flow systems), and temperature control in optimizing the reaction efficiency. The article concludes by integrating these concepts into a comprehensive framework for optimizing photoiniferter and PET-RAFT polymerizations.

## Graphical abstract

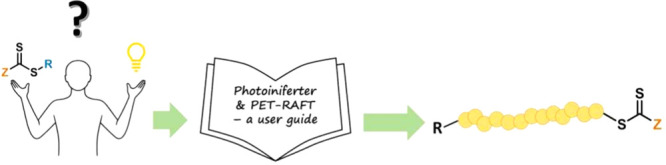

## 1 Introduction

Reversible deactivation radical polymerization (RDRP) techniques have changed the landscape of polymer chemistry, enabling the synthesis of polymers from a diverse range of functional monomers with narrow molecular weight distributions, highly complex polymeric architectures, well-defined end-groups, and functionalities. One of the most widely used RDRP methods is reversible addition–fragmentation chain-transfer (RAFT) polymerization due to its ease of implementation and remarkable tolerance toward functional groups and reaction conditions. There has been an ongoing effort to activate RAFT polymerizations with nonthermal initiation methods. Among these are redox chemistry,^1^ mechanical,^2,3^ and electrochemical methods,^4–6^ enzyme-mediated pathways,^7^ and light activation.^8–10^ The low cost and ubiquity of light, as well as the spatiotemporal control it can provide, position light activation among the most attractive alternative modes of activation of RAFT polymerization. In fact, decades before RAFT polymerization was invented in 1998,^11–13^ Takayuki Otsu and co-workers used thiocarbonylthio compounds to prevent irreversible termination of growing polymer chains to achieve controlled photopolymerization termed iniferter polymerization.^14,15^ Within the realm of photopolymerizations mediated with thiocarbonylthio compounds, we can distinguish: (1) photoRAFT in which initiating radicals are generated from the decomposition of a photoinitiator, (2) photoiniferter polymerization which relies on direct photoexcitation of the iniferter, and (3) photoinduced energy/electron transfer RAFT (PET-RAFT) which involves a photocatalyst (PC). Note that photoiniferter polymerization is often incorrectly referred to as “photo-RAFT” or “photoiniferter-RAFT”. Although degenerative chain transfer forms a part of the photoiniferter mechanism, the photoiniferter is not a variant of RAFT but a distinct type of polymerization. Herein, we follow terminology outlined by Sumerlin at al. in their recent review.^16^

Photopolymerizations offer many advantages, such as spatiotemporal control, oxygen tolerance, orthogonality, initiator-free polymerization, low reaction temperature, and more, and as such, they form a powerful toolbox for delivering high-precision materials for advanced applications. The development and applications of photopolymerizations mediated with thiocarbonylthio compounds have been the subject of multiple reviews.^17–22^ They have found applications in light-activated self-healing materials,^23,24^ 3D printing,^25–29^ surface coating,^30,31^ and more. The absence of initiator-derived chains in photoiniferter and PET-RAFT polymerizations aids the preparation of sequence-controlled polymers such as the precise addition of single monomer units,^32–35^ as well as unprecedented well-defined ultrahigh molecular weight polymers,^36–38^ including star polymers^39^ and bottlebrushes,^40^ with minimal bimolecular termination. The wavelength selectivity of photoRAFT can be used to prepare nano-objects with high-order morphologies via polymerization-induced self-assembly (PISA).^41^ Oxygen-tolerance of such systems enables the synthesis of polymer libraries in a high-throughput manner.^42–44^

Herein, the features and limitations of photopolymerizations mediated with thiocarbonylthio compounds, particularly photoiniferter and PET-RAFT, are summarized, highlighting the strengths and weaknesses of both systems. All aspects are considered from a practical point of view to create a guide to successful polymerizations. Fundamental aspects of RAFT polymerization and photochemistry of thiocarbonylthio compounds are covered to lay the foundation on which practical elements, such as choice of RAFT agent and catalyst, wavelength and intensity of light, and reactor design, are discussed.

## 2 Conventional RAFT Polymerization—Mechanism and Choice of RAFT Agent

In RAFT polymerization, the equilibrium between active and dormant chains is achieved by degenerative chain transfer facilitated by a chain transfer agent (CTA/RAFT agent), which is typically a thiocarbonylthio compound (ZC­(=S)­SR) with various R and Z groups (Scheme 1). Contrary to RDRP systems based on reversible deactivation, such as nitroxide-mediated radical polymerization (NMP)^45,46^ and atom transfer radical polymerization (ATRP), a constant supply of radicals is needed because the activation–deactivation process does not alter the absolute number of radicals in the system, nor does it prevent termination. The mechanism of RAFT is similar to free radical polymerization, with degenerative chain transfer occurring in parallel to the propagation and termination steps (Scheme 1).

**Scheme 1:**
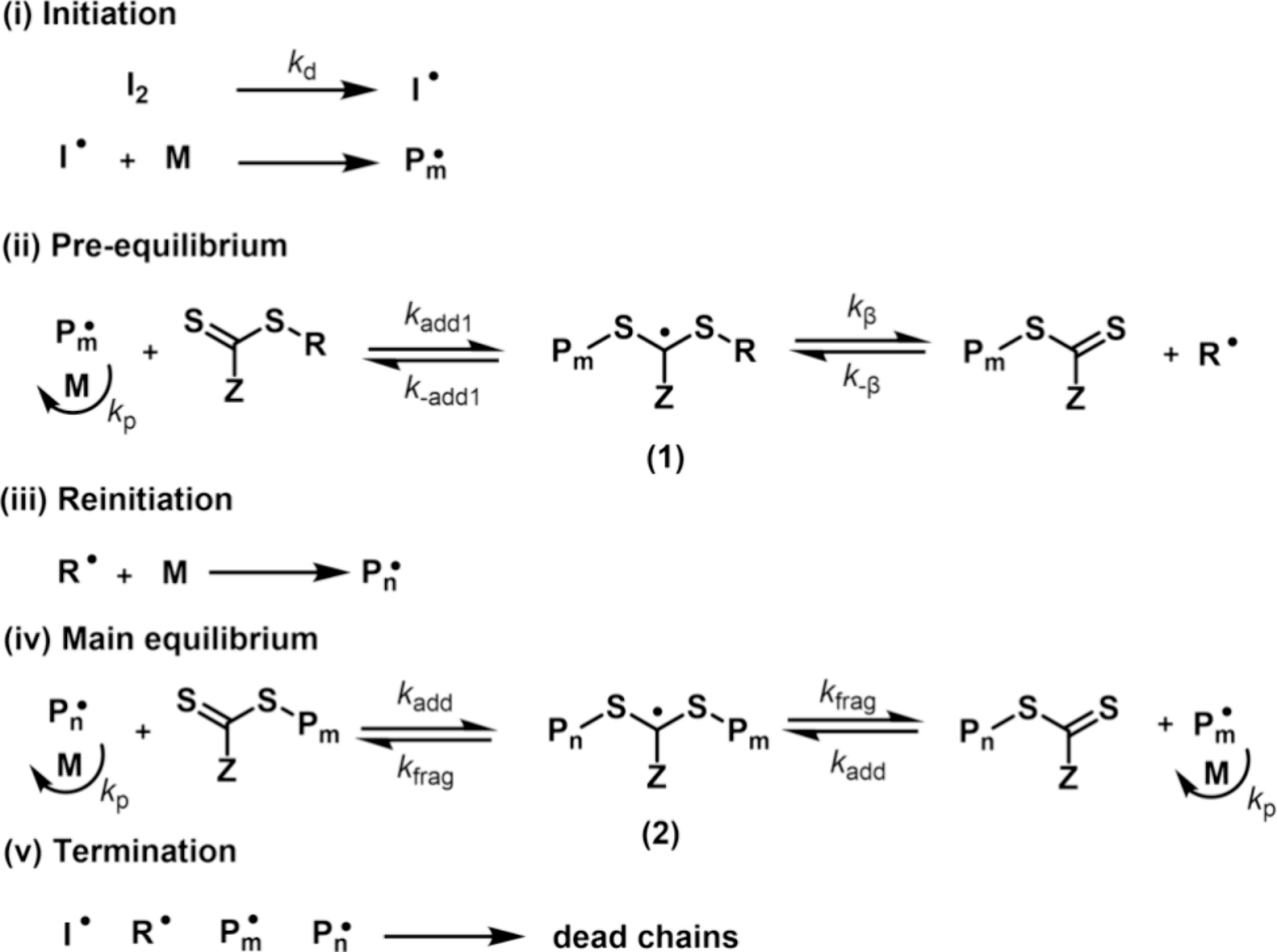
Proposed Mechanism of RAFT Polymerization

Following decomposition of an initiator molecule (step i), the resulting radicals react with monomers to create macroradicals that add to the RAFT agent (step ii) and enter the RAFT pre-equilibrium-fragmentation leading to a radical, the (R^•^) capable of initiating polymerization. In RAFT polymerization, the majority of polymer chains are initiated from R groups. In fact, the number of chains impacted by termination events depends on the number of chains initiated with the initiator fragment, as this reduces the ratio of thiocarbonylthio groups to growing chains in the degenerative chain transfer equilibrium.^47^ Hence, keeping the initiator concentration low is crucial if high end-group fidelity is required. Once all the original RAFT agent has been converted to macroCTA, the system is in the main RAFT equilibrium (step (iv)), which allows for fast exchange between dormant and active states, leading to near-uniform growth of polymer chains. This equilibrium is governed by two rates: the rate of addition of a macroradical (active chain) to macro-CTA and the rate of fragmentation of the RAFT intermediate 2. When the propagating radicals are in the active state, they can terminate, like in free radical polymerization (FRP), via either combination or disproportionation. However, unlike in FRP, where chains are either active or terminated, in RAFT polymerization, the chains spend most of the time in the dormant state, and hence, the fraction of chains subject to termination is lower. For RAFT polymerization to be effective, i.e., leading to uniform growth of polymers, the addition–fragmentation rate coefficients should be higher than that of propagation; assuming ideal behavior, polymer chains undergo many activation–deactivation cycles with only a few monomers added per cycle, representing overall a small fraction of the target chain length.^48^

The radical stability of the propagating chain end should match both the reactivity of the C=S double bond of the CTA (as controlled by the Z-group) and the radical stability of the R-group. The C=S bond of the RAFT agent needs to be more reactive than the C=C bond of the monomer toward radical addition. The reactivity of the C=S bond is controlled by the Z group and decreases in order: aromatic dithioesters (dithiobenzoates), trithiocarbonates, xanthates, and dithiocarbamates (Figure 1). For the last two groups, the resonance contribution from the lone pair of the heteroatom (nitrogen or oxygen) reduces the C=S double bond character by increasing the electron density, rendering the C=S bond less reactive toward radical addition. The reactivity of dithiocarbamates depends on the substituents on the nitrogen—if the nitrogen lone pair is not available to interact with C=S double bond because if forms part of an aromatic ring (such as pyrrole or carbazole) or a conjugated system (such as pyrrolidinone), the corresponding dithiocarbamate is more reactive.^49^ The R group has a minimal effect on the rate of addition to thiocarbonylthio compound, but it governs partitioning of the intermediate radical between starting materials and products (reaction 2, Scheme 1). For the RAFT process to be effective, the degenerative chain transfer equilibrium must be established rapidly, requiring fast fragmentation of the intermediate radical (species 1).The R group needs to be a better homolytic leaving group than the propagating radical, i.e., more stable R groups will fragment more efficiently. However, the R group also needs to reinitiate polymerization, which means steric factors and polarity play an important role.^50^ Slow fragmentation of the intermediate RAFT radical (Scheme 1, 1) and insufficient reinitiating ability of the RAFT leaving group (R) radical often lead to an induction period, defined as the time where no or minimal monomer conversion occurs before complete consumption of the RAFT agent.^51^ However, in an ideal scenario, the CTA is consumed early, and the pre-equilibrium does not affect the main RAFT equilibrium that governs control over polymer growth.

**Figure 1:**
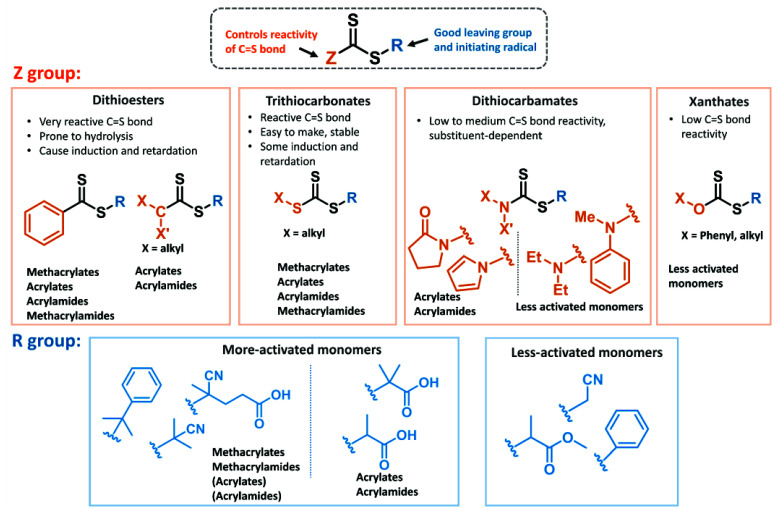
Guideline for selection of appropriate RAFT agent (R and Z groups) for various monomers.

Therefore, correct monomer/RAFT agent matching is essential for controlled polymer growth. Typical monomers polymerized with RAFT can be divided into more-activated monomers (MAMs) and less-activated monomers (LAMs). MAMs form more stable propagating radicals as their double bond is conjugated, typically to an aromatic ring or carbonyl group; examples include styrene, (meth)­acrylates, and (meth)­acrylamides. Substitution of the double bond with a methyl group makes the radical derived from the monomer more stabilized, making the monomer overall more reactive – e.g., methacrylates are more reactive than acrylates.^52^ In LAMs, the double bond is typically substituted with a heteroatom-based group (oxygen in vinyl acetate or nitrogen in *N*-vinylpyrrolidone). MAMs perform best with RAFT agents with a strong C=S double bond, such as dithioesters (especially aromatic dithioesters - dithiobenzoates, Z = Ph) and trithiocarbonates (Z = S-alkyl). LAMs, due to the lower stability of their radicals and poor leaving group ability, require less stable intermediate RAFT radicals to favor fragmentation and can be controlled with xanthates (Z = O-alkyl) and dithiocarbamates (Z = *N*-alkyl). Lower reactivity MAMs (i.e., acrylates and acrylamides) can be controlled with xanthates and dithiocarbamates when an appropriate substituent on the heteroatom is chosen. The reactivity of CTA can be further tuned by the R group. Methacrylates and methacrylamides require a tertiary R group with either aromatic or cyano substituents (such 2-cyano-2-propyl and 4-(4-cyanopentanoic acid))­for the best control, while acrylates and acrylamides work best with a secondary R group (such as 3-(propanoic acid)), a benzyl group, or a tertiary group, such as 2-(2-methylpropanoic acid). LAMs, on the other hand, work well with secondary, cyanomethyl or benzylR groups (Figure 1).

## 3 Photopolymerizations Mediated with Thiocarbonylthio Compounds

Three distinct mechanisms of photopolymerizations mediated with thiocarbonylthio compounds are photoRAFT, photoiniferter, and photoinduced energy/electron transfer RAFT (PET-RAFT) polymerization. PhotoRAFT is analogous to conventional thermal RAFT in which the thermal initiator has been replaced with a photoinitiator that decomposes and generates radicals upon activation with a particular wavelength of light.^10^ Photoiniferter polymerization utilizes the intrinsic photochemistry of thiocarbonylthio compounds (iniferters) to undergo photolysis upon exposure to light and act as both initiator and chain transfer agent, and reversible terminator.^8,9^ In PET-RAFT, upon activation with light, an exogenous catalyst undergoes an energy or electron transfer to the RAFT agent ultimately leading to its fragmentation.^53,54^

### 3.1 Photoinitiated RAFT

In photoRAFT, a conventional thermal initiator is replaced with a photoinitiator/photosensitizer that generates reactive species (free radicals or ions) that initiate polymerization. The photoinitiator can undergo homolytic bond cleavage to generate free radicals (type I photoinitiator). Alternatively, active species are generated via a bimolecular pathway via hydrogen abstraction of electron transfer reactions between a photosensitizer and a co-initiator (type II photoinitiator).^55,56^ Most commercially available photoinitiators are activated by ultraviolet (UV) or high-energy visible light such as violet and blue wavelengths. Photoinitiators usually contain aromatic rings to enhance light absorbance, and typical sensitizers include alcohols, amines, and thiols. For a list of commonly employed unimolecular and bimolecular initiators, the reader is directed to the review by Pan et al.^57^

While the use of a photoinitiator enables polymerizations at milder conditions, such as at lower temperatures, and hence reduces branching and irreversible chain transfer events, the polymerization follows the conventional RAFT mechanism and, as such, will be similarly affected by the contamination of end groups caused by the imbalance between the number of polymer chains and thiocarbonylthio moieties available due to initiation of new chains by the initiator fragments. Further, if the wavelength required for activation of the photoinitiator coincidentally excites the RAFT agent, which is the case for the majority of UV-active initiators, a secondary initiation pathway is introduced (photoiniferter mechanism), and photodegradation of the polymer end group is possible. Hence, photoinitiators are most commonly used in free radical polymerization, and their use and optimization in RAFT polymerization are outside the scope of this review. For an overview of recent developments in photoinitiator systems, the reader is directed to the reviews by Yagci et al.^58^ and Shao et al.^59^ Briefly, photoinitiator efficiency depends on quantum yield for dissociation, addition of coagent (type II photoinitiator), wavelength of light used^60^ as well as its intensity, tolerance to side reactions, and reactivity toward monomer.^61^

### 3.2 Photoiniferter Polymerization

The photoiniferter process was first reported by Otsu and co-workers before the discovery of RAFT polymerization, in which an iniferter compound acts simultaneously as an *ini*tiator, trans*fer* agent, and *ter*minator.^62,63^ Typical iniferter compounds include dithiocarbamates, which possess labile C–S or S–S bonds that can decompose either thermally or upon exposure to light. Upon decomposition, the iniferter fragments can initiate polymerization and reversibly combine with the growing polymer chains, thus controlling their growth. Chain transfer agents typically employed in RAFT polymerization can act as iniferters. In photoiniferter polymerization, the CTA, upon excitation with either UV^10^ or visible light,^8,9^ undergoes a homolytic C–S bond cleavage to release a C-centered R-group radical and a thiyl radical (Scheme 2). The leaving group radical initiates polymerization, and the thiyl radical can reversibly combine with the propagating macroradicals, which can then enter degenerative chain transfer equilibria. In the seminal iniferter work reported by Otsu and co-workers, reversible combination was the dominant mechanism to control polymer growth^14,64,65^ due to poor mismatch of monomer and iniferter reactivities; however, with well-matched thiocarbonylthio end group, excellent control over molecular weight distribution can be achieved. The exchange between active and dormant states must be significantly faster than propagation to ensure polymer chains grow at similar rates.

**Scheme 2:**
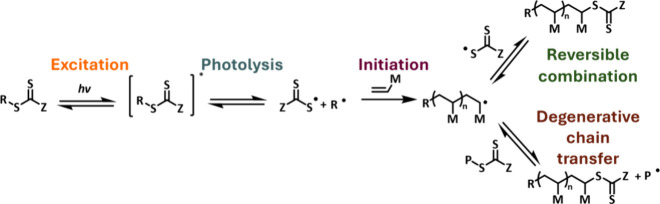
Proposed Mechanism of Photoiniferter Polymerization

The interplay of reversible combination and degenerative chain transfer depends on the nature of the iniferter, reactivity of the monomer, and reaction conditions. Trithiocarbonates with higher *C*_tr_ and slower photolysis are more likely to participate in degenerative chain transfer than xanthates, which undergo rapid photodissociation. Reversible combination is more challenging to control, particularly for more stable propagating radicals the exchange rate provided by reversible combination may be insufficient for effective exchange between polymer chains. Still, there are examples in which excellent molar mass control and low dispersities can be achieved. One example is the use of xanthates for the production of ultrahigh molecular weight polymers via photoiniferter polymerization.^38^ However, in other cases the activation–deactivation equilibrium was found to be insufficient except when propagation was artificially slowed by slow or sequential monomer addition.^66^ However, reversible deactivation provides a significant advantage in block copolymer synthesis. Typically, in RAFT polymerization, a MAM block needs to precede a LAM block (for example, a methacrylic block needs to be prepared first, followed by an acrylic segment) to ensure efficient fragmentation of the macroCTA. However, in photoiniferter polymerization this monomer sequence can be reversed, and this approach worked for xanthate or dithiocarbamates RAFT agents but was less effective for trithiocarbonates as the role of degenerative chain transfer for the latter was too significant.^67^

In conventional RAFT polymerization, the number of termination events is proportional to the number of chains initiated with an initiator fragment. Hence, keeping the CTA to initiator ratio low is one of the prerequisites for high livingness of RAFT polymerization. In the photoiniferter process­(as in the case of PET-RAFT, see below) there is no exogenous source of radicals; so theoretically, irreversible termination events can be completely excluded. In the absence of short initiator-derived macroradicals, polymer chains are unlikely to undergo bimolecular termination due to limited diffusion in viscous media. Although termination events cannot be completely excluded for reasons discussed later in this review, photoiniferter polymerization was nonetheless shown to provide high levels of control for various monomer families, and well-defined polymers with unprecedented high molecular weight were prepared with photoiniferter polymerization.^38,68,69^

### 3.3 PET-RAFT

PET-RAFT was first reported by Boyer and co-workers and relies on excitation of a photocatalyst that, in the excited state, can interact with a RAFT agent to generate initiating carbon-centered radicals.^53^ Contrary to photoRAFT polymerization, the photosensitizer is not incorporated into the polymer chains, as the R group of the RAFT agent acts as the exclusive initiating species. There are two proposed mechanisms of radical generation in PET-RAFT—photoinduced energy transfer or photoinduced electron transfer (PET) (Scheme 3).^54,70^ In photoinduced energy transfer, the excited state photocatalyst transfers its energy to the CTA, facilitating homolysis or fragmentation of the CTA, in a manner analogous to the photoiniferter mechanism. In this way, photoinduced energy transfer RAFT can be thought of as a photocatalyzed photoiniferter process. In contrast, photoinduced electron transfer, the photocatalyst in the excited triplet state, can undergo oxidative or reductive quenching by the thiocarbonylthio compound or a cosensitizer (electron donor) to reduce the thiocarbonylthio compound to a radical anion, which then fragments to generate a propagating radical. Both pathways are possible since many photoredox catalysts have a high-energy electron and a low-energy vacant orbital. The resulting propagating radical can enter the degenerative chain transfer equilibrium and form a dormant polymer chain. Regeneration of the catalyst via electron/energy transfer closes the catalytic cycle. For the CTA to effectively quench the excited state photocatalyst, the redox potential of the thiocarbonylthio compound needs to be higher than the reduction potential of the photocatalyst.^53^ Further, the redox process must be efficient to ensure fast and complete regeneration of the photocatalyst.^71^ The nature of the photocatalyst, wavelength of light, and presence of electron donors dictate which mechanism takes place, with a complex interplay of mechanisms being possible.^72^ However, the exact mechanism remains ambiguous for every combination of monomer, CTA, and photocatalyst.^70,73^ For a detailed mechanistic perspective on PET-RAFT, the reader is directed to the review by Allegrezza et al.^54^

**Scheme 3:**
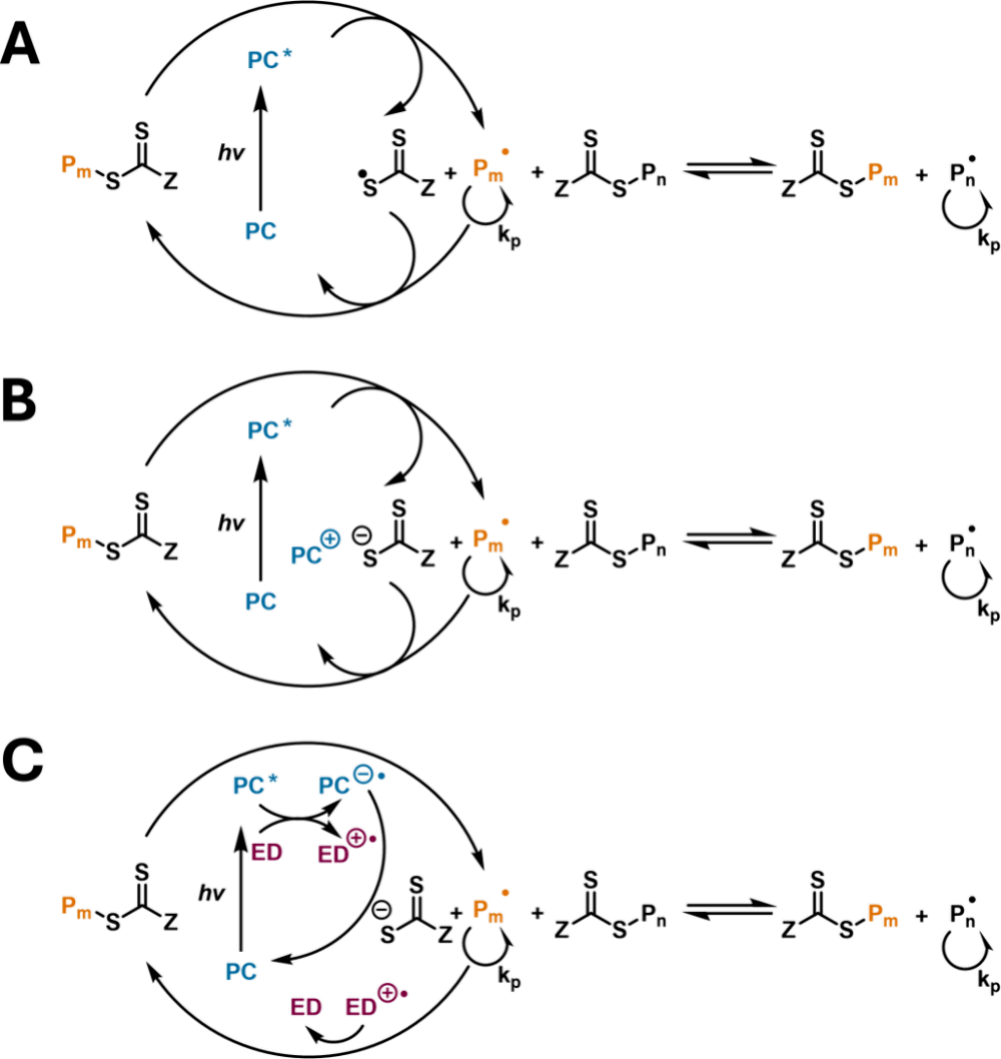
Different Mechanisms of PET-RAFT: (A) Photoinduced Energy Transfer Mechanism, (B) Photoinduced Oxidative Electron Transfer Mechanism, and (C) Photoinduced Reductive Electron Transfer Mechanism^a^

a PC: Photocatalyst. ED: Electron donor.

#### 3.3.1 Choice of Catalyst

Typical photoredox catalysts employed in PET-RAFT involve transition metal complexes based on iridium^53^ such as tris­[2-phenylpyridine]­iridium­(III) (Ir­(ppy)_3_) or ruthenium^74^ such as tris­(bipyridine)­ruthenium­(II)­chloride ([Ru­(bpy)_3_]­Cl_2_) (Figure 2) which possess high molar absorption coefficients and have been widely used in photo-organic chemistry. [Ru­(bpy)_3_]­Cl_2_ has the added advantage of a good water solubility. Metalloporphyrins - catalysts based on more abundant metals, in particular Zn tetraphenylporphine (ZnTPP), are good alternatives for PET-RAFT polymerization.^75^ Moreover, ZnTPP has the advantage of working with longer light wavelengths, such as green, orange, and red, while iridium- and ruthenium-based catalysts work with higher energy light, such as violet and blue. Among metalloporphyrins with different metal centers, ZnTPP was found most suitable for PET-RAFT polymerization, while Fe and Co and Ni metalloporphyrins (FeTMPP, CoTMPP, NiTPP) resulted in no or sluggish polymerization under red light due to limited metal to ligand charge transfer.^75^ Metal-free catalysts, such as organic dyes like fluorescein, erythrosine, and Eosin Y,^76,77^ or porphyrins, such as Pheophorbide A,^78^ have also been shown to be effective for PET-RAFT processes. Nature-abundant chlorophyll pigments, such as chlorophyll a (Chl a), are also promising alternatives to metal-based catalysts, albeit they are prone to degradation and highly oxygen-sensitive.^79^ Heterogenous catalysts, such as Eosin Y grafted silica nanoparticles,^80^ and inorganic metal oxides such as zinc oxide (ZnO)^81^ and titanium dioxide (TiO_2_),^82^ as well as plasmonic nanoparticles^83,84^ have also been successfully adapted for PET-RAFT polymerizations, and their high chemical stability, low cost, and easy removal by filtration make them promising alternatives to transition metal complexes. While there are many photocatalysts to choose from, they are not without limitations. New catalysts need to be developed to improve polymerization rates and broaden the range of RAFT agents that can be effectively activated. The examples and principles guiding photocatalyst design for photocontrolled polymerizations are covered in detail elsewhere.^85^

**Figure 2:**
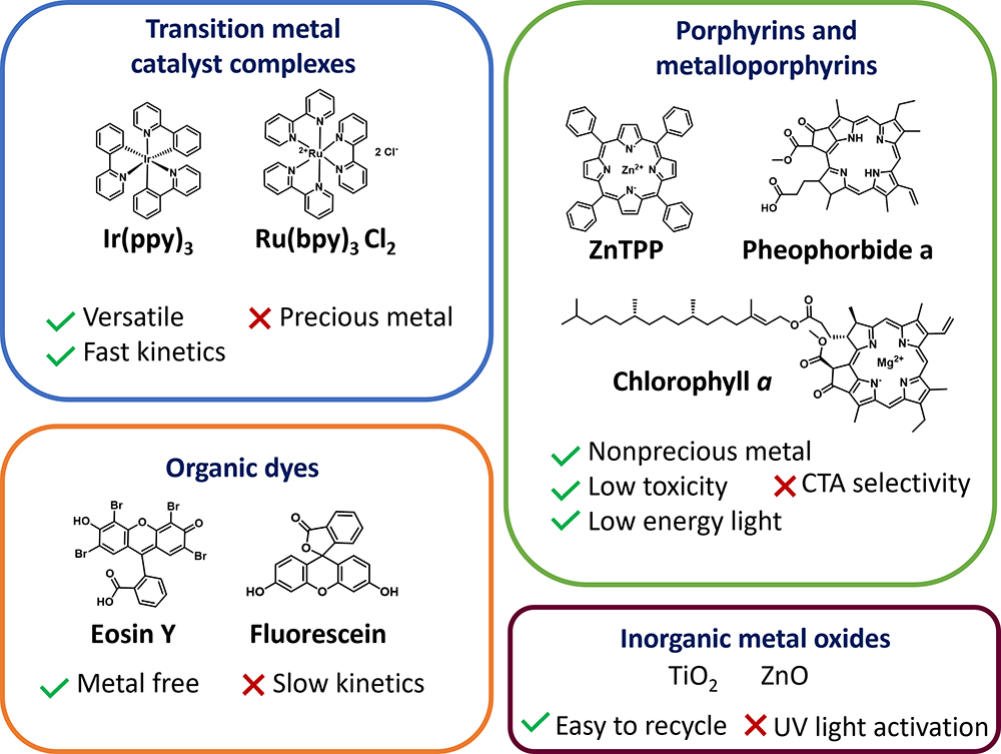
Catalyst commonly used in PET-RAFT polymerization.

Monomer reactivity does not directly impact the choice of photocatalyst; however, some catalysts have been shown to selectively activate certain RAFT agent families.^75,78,86^ While the exact mechanism of this selectivity is not yet known and will depend on the catalyst–RAFT agent pairing, for the catalysts that participate in energy transfer, this is likely linked to energy proximity of the excited orbital of the photocatalyst and the charge transfer orbital.^86^ Notable examples of catalyst selectivity include meso-tetraphenylporphyrin (TPP), which works with dithiobenzoates, but leads to sluggish polymerization with trithiocarbonates.^75^ On the contrary, ZnTPP exhibits unusual selectivity and works with trithiocarbonates that are more difficult to activate due to higher redox potential, but not with dithiobenzoates, dithiocarbamates, or xanthates.^75^ Pheophorbide A (PheoA) was also found to work selectively with 4-cyanopentanoic acid dithiobenzoate but not with other dithiobenzoates or xanthates and trithiocarbonates, and this phenomenon was used to prepare bottlebrush polymers by selectively polymerizing methacrylic backbone with PheoA followed by activation of pendant trithiocarbonate to graft acrylic arms.^78^ This selectivity is associated with the activation of the RAFT agent and no selectivity is observed when macroCTA is used.^86^

While the typical catalyst loading used in PET-RAFT polymerizations is low (in the ppm range) catalyst removal may be necessary for specific applications such as biomedical applications due to the toxicity of many metal catalysts, particularly transition metal complexes, which is one of the significant limitations of PET-RAFT. While the catalyst can be immobilized on a solid support, such as silica particles^80^ or cellulose,^87^ to aid removal and recycling, the catalyst is eventually deactivated and cannot be reused beyond a few cycles.^88^

## 4 Photochemistry of Thiocarbonylthio Compounds

The UV–vis absorption spectra of the structurally diverse thiocarbonylthio compounds (Figure 3) are primarily determined by their Z-groups. Two absorption bands corresponding to π–π* and n−π* transitions are observed, and the position and intensity of these bands vary depending on the RAFT agent’s structure. Thiocarbonylthio compounds exhibit a range of solvatochromism, from low for trithiocarbonates to moderate for dithiobenzoates.^89,90^ For trithiocarbonates and dithioesters, the π–π* transition occurs around 300 nm (UV range), while the π* transition lies in the visible light spectrum. The n−π* transition is red-shifted in dithioesters (λ_max_ ∼ 500 nm) compared to trithiocarbonates (λ_max_ ∼ 430–445 nm), giving those compounds red and yellow color respectively.^89^ In contrast, for dithiocarbamates and xanthates, the n−π* transition is significantly blue-shifted (350 nm) due to increased electron density at the C=S bond.^38^

**Figure 3:**
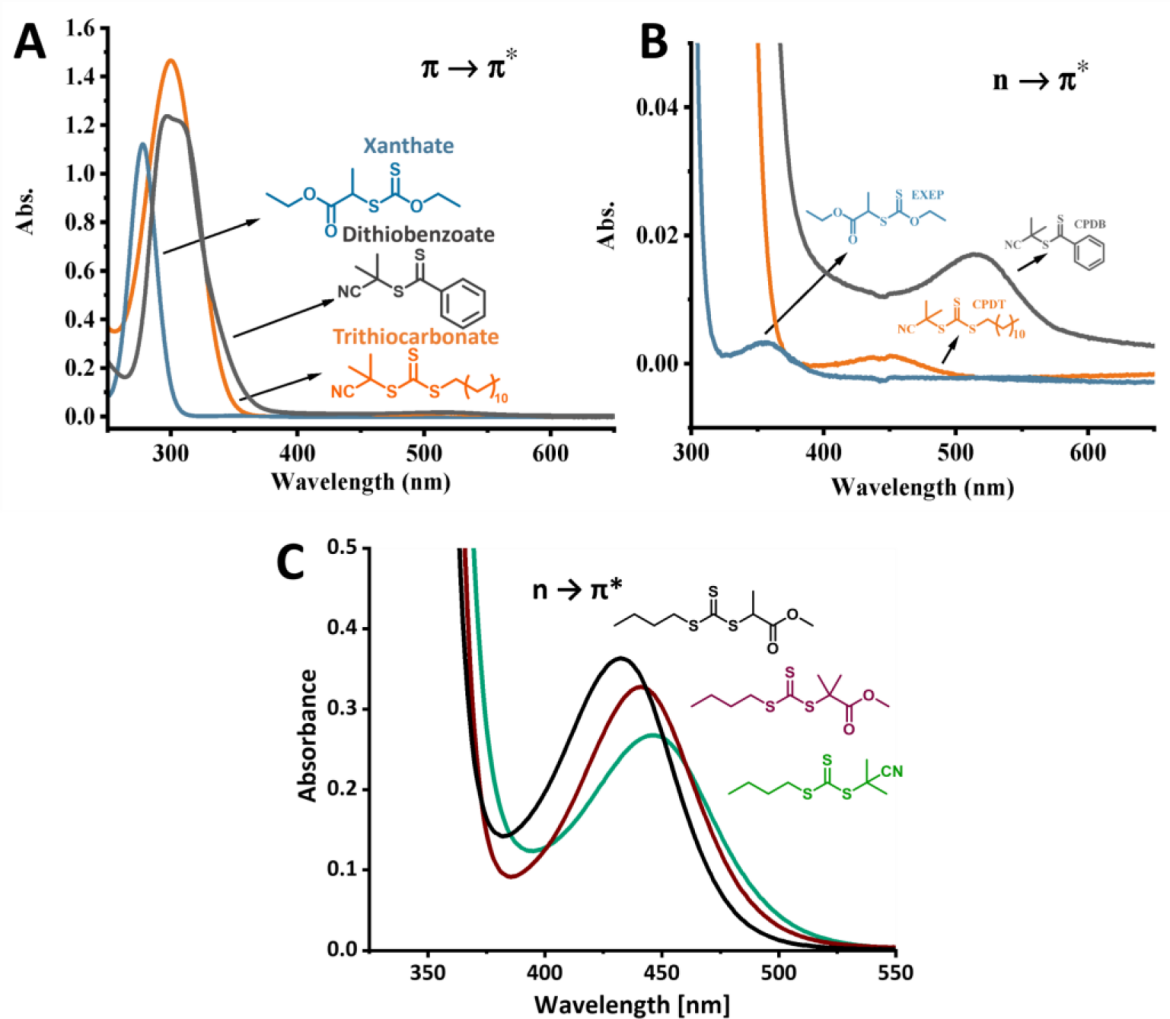
UV–vis absorption spectra of different thiocarbonylthio compounds: (A, B) in ethyl acetate, 1 mM concentration, (C) in DMSO, 10 mM concentration. Adapted with permission under a Creative Commons CC BY 4.0 License from Li, J.; Zhang, M.; Zhu, J.; Zhu, X. *Polymers* **2019**, *11*(10), 1722.^115^ Copyright 2019 Li, J.; Zhang, M.; Zhu, J.; Zhu, X.

Although the R-group also affects the UV–vis absorption spectrum, the contribution is limited. For trithiocarbonates, tertiary R groups lead to a ∼10 nm red shift relative to secondary alkyl and benzylic R groups (Figure 3). As a symmetry-forbidden transition, the n−π* transition has a significantly lower molar absorption coefficient than the allowed π–π* transition, with typical values for trithiocarbonates of ε = 15–120 L mol^–1^ cm^–1^ and 8000–20000 L mol^–1^ cm^–1^, respectively.^89^

Thiocarbonylthio compounds have three readily available excited states: a higher-energy singlet state, S2 (corresponding to π–π* transition), and a lower-energy first singlet S1 and first triplet T1 states (n−π* transition).^91^ According to Kasha’s rule, photochemical transformations typically occur from the lowest excited state of a given multiplicity, regardless of the excitation wavelength.^92^ However, due to a large energy gap between S1 and S2, thiocarbonylthio compounds can violate this rule, with different light wavelengths populating distinct excited states.^93^ This large gap also makes the S2 excited state relatively long-lived,, as opposed to short-lived S1 state which easily passes to T1 state via intersystem crossing the due to a small energy gap between those two states.^94^ As a result, photochemical transformations where the RAFT agent is excited directly, like in iniferter polymerization, happen from the S2 and T1 excited states, while in PET-RAFT, the RAFT agent is excited to the T1 state via energy/electron transfer from the photocatalyst in its T1 state.

The relationship between the excited states and the rate of photolysis of the RAFT agents remains poorly understood. The quantum yield of dissociation, which indicates the efficiency of photochemical transformation by comparing the number of photoinduced changes to the number of photons absorbed, does not correlate directly with the absorbance spectrum. Higher molar absorption coefficients do not necessarily lead to faster photolysis. For thioketones, the quantum yield for homolytic bond cleavage is typically higher for the n−π* transition, despite a lower molar absorption coefficient compared to the π–π* transition.^91^

For a bond cleavage to occur, the excited state must have an energy exceeding the dissociation energy of the bond. The C–S bond dissociation energy depends not only on the Z group but also on the R group—the more stable the R group radical, the lower the bond dissociation energy.^95–97^ For trithiocarbonates, all three excited states (S1, T1, and S2) typically have sufficient energy to lead to homolytic C–S bond cleavage by β-scission, allowing activation with either UV or visible light.^98^ Dithiobenzoates, on the other hand, have low-lying S1 and T1 energy levels, and excitation into those states should not provide enough energy to break the C–S bond,^98^ yet successful photoiniferter polymerization mediated with dithiobenzoates under blue light was reported.^99^ Xanthates, with both lower bond dissociation energies and a significantly higher molar absorption coefficient for the n−π* transition, exhibit increased photolysis rates.^100^

In general, the homolytic bond cleavage of thiocarbonylthio compounds tends to have low quantum yields, which can result from efficient and fast recombination after photolysis or fast relaxation of excited states. The stability of the R group dictates the lifetime of the radical and will affect the rate of recombination of the C- and S-centered radicals, leading to an apparent diminished rate of photolysis. However, the increased steric hindrance of tertiary R groups leads to their slower rate of recombination. Overall, RAFT agents with more stable R groups have higher rates of photolysis. The process is further affected by the presence and nature of the monomer. The following section will address the practical aspects of the RAFT agent photochemistry, focusing on how to select appropriate light wavelengths to maximize polymerization rates while minimizing photodegradation and loss of control over the polymerization process.

## 5 Choosing the Correct Wavelength of Light

### 5.1 PET-RAFT Polymerization

In PET-RAFT polymerization, the choice of the wavelength of light is dictated by the catalyst. The majority of the commonly used catalysts, such as transition metal complexes Ru­(bpy)_3_Cl_2_ ^101^ and Ir­(ppy)_3_ ^102^ work under low irradiance blue light (typically low mW/cm^2^, Figure 4). PET-RAFT photocatalysts are generally more photoactive than RAFT agents, allowing PET-RAFT to be effectively used with lower irradiance values compared to those typically required in photoiniferter polymerization. An iridium-based catalyst was also shown to work effectively under violet and green light.^102^ Activation with green or lower energy light is particularly appealing as it further decreases the likelihood of direct photodissociation of the RAFT agent and should lead to improved polymerization control as the photoiniferter activation is excluded. Eosin Y-catalyzed photoinduced polymerization was shown to proceed via a number of different initiation pathways, depending on the wavelength of light used (blue, 450 nm vs green, 520 nm), and the addition or absence of sacrificial reducing agent (e.g., tertiary amine or ascorbic acid).^72^ Green light was found to be the most selective, and in the absence of a tertiary amine, oxidative PET-RAFT mechanism occurred exclusively, leading to fast polymerization kinetics and superior molecular weight control.

**Figure 4:**
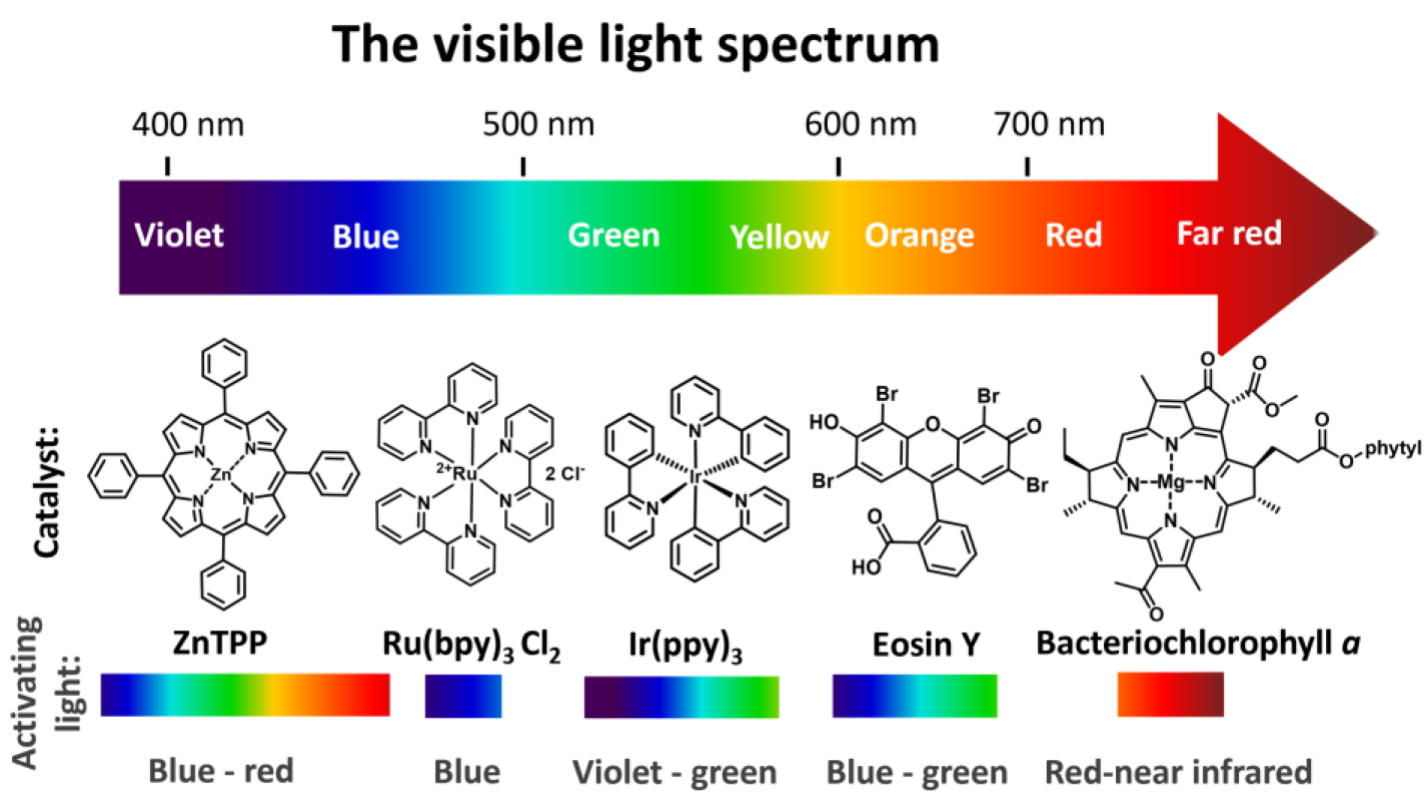
Commonly employed a photocatalyst in PET-RAFT polymerization and compatible wavelengths of light.

Metalloporphyrins usually have multiple absorption maxima and can work under a range of different light wavelengths. Zinctetraphenylporphinewas found to work efficiently under wavelengths ranging from 435 to 655 nm, and similar levels of control were observed for all tested wavelengths.^75^

To further improve selectivity and light penetration, chlorophyll based catalysts were explored, as they absorb red light.^103^ Bacteriochlorophyll a (BChl a) was found to be compatible with red (635 nm), far-red (780 nm), and near-infrared (850 nm) light,^104^ while chlorophyll a (Chl a) was also shown to be capable of driving polymerization under red (635 nm) and blue (461 nm) but not green (530 nm) light.^79^ It was found that polymerization under red light proceeded faster and with a shorter induction period despite a lower absorption coefficient at 665 nm compared to the 430 nm band, likely due to competition for light between the RAFT agent and the catalyst under blue light irradiation. While majority of PET-RAFT catalysts work under visible light, many near-infrared active compounds (such as, phthalocyanine, naphthalocyanine) have been adopted for polymerization.^105^

The rate of polymerization can be tuned with the wavelength of light for multiple catalysts. For similar light intensities, the rate of polymerization of methyl acrylate with iridium catalyst was found to increase in order green (510 nm, 6.4 mW/cm^2^) < blue (450 nm, 5.4 mW/cm^2^) < violet (399 nm, 5.4 mW/cm^2^) light (Figure 5).^102^ The induction period was also found to be the longest for green light. Similarly, polymerization of MA with ZnTPP was fastest at 565 nm light, a wavelength that overlaps closely with the most intense absorption maximum of the catalyst at 570 nm. Shorter (522 nm) and longer (595 nm) wavelengths led to slower polymerizations.^75^

**Figure 5:**
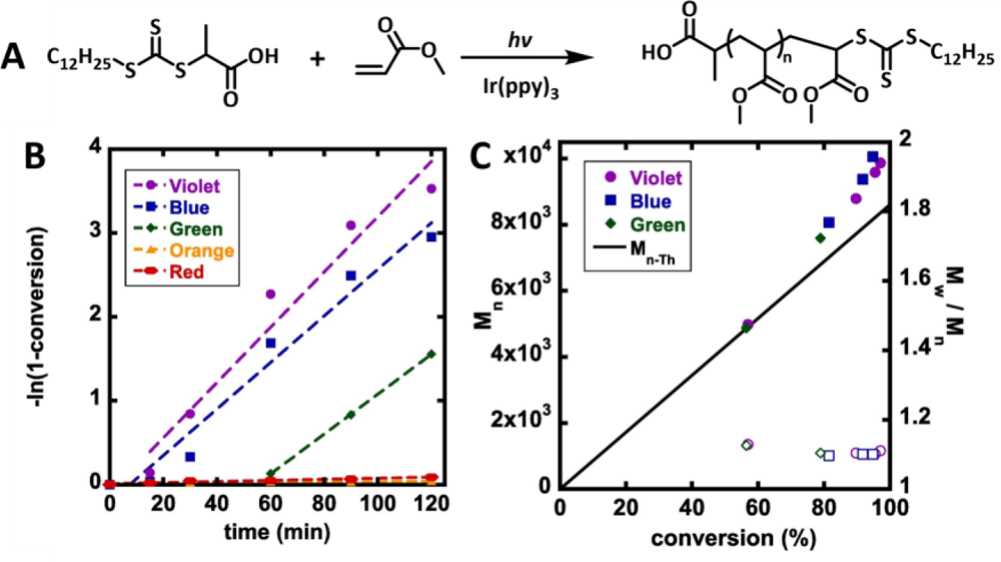
(A) PET-RAFT polymerization of MAwith Ir­(ppy)_3_under different wavelengths of light. (B) Kinetics of polymerization under violet (399 ± 8 nm, 5.4 ± 0.2 mW/cm^2^), blue (450 ± 10 nm, 5.4 ± 0.2 mW/cm^2^), green (510 ± 20, 6.4 ± 0.5 mW/cm^2^), orange (591 ± 7 nm, 1.1 ± 0.1 mW/cm^2^), and red (630 ± 10 nm, 4.6 ± 0.5 mW/cm^2^) light. (C) Evolution of *M*_n_ (solid points) and *M*_w_/*M*_n_ (hollow points) with monomer conversion. The solid line represents the theoretical *M*_n_ values. Reprinted from Parnitzke, B.; Nwoko, T.; Bradford, K. G. E.; De Alwis Watuthanthrige, N.; Yehl, K.; Boyer, C.; Konkolewicz, D. Photons and Photocatalysts as Limiting Reagents for PET-RAFT Photopolymerization. *Chemical Engineering Journal* **2023**, *456*, 141007.^102^ Copyright 2023 with permission from Elsevier.

### 5.2 Photoiniferter Polymerization

In photoiniferter polymerization, selecting the appropriate light wavelength is critical for polymerization’s success. The wavelength of light as well as the light intensity affect the photolysis rate, which, in turn, affects initiation and the contribution of reversible combination to the polymerization mechanism. While the absorbance spectrum of a RAFT agent hints toward potential activation wavelengths, the information it provides is limited. Frequently in photochemistry, the most activating wavelength is red-shifted relative to the absorption maximum.^106–109^ Photochemical reactivity at different wavelengths can be probed by studying photochemical action plots obtained with monochromatic tunable laser systems which allow photoexcitation of molecule of interest with stable number of photons at each wavelength.^110^ Such system was applied by Nardi et al. to study wavelength-resolved photochemically induced copper-mediated polymerization of methyl acrylate between 305 and 550 nm.^106^ A red shift of the action plot compared to the copper­(II) catalyst absorption spectrum was observed, and the wavelength of light was found to impact conversion, number-average molecular weight, and dispersity of the resulting polymers. Similar findings were reported for a wider range of monomers in a study by Ma et al. using LEDs with narrow emission spectra.^111^ While detailed study of thiocarbonylthio mediated photopolymerization action plots is lacking, studies employing LEDs showed similar trends.^112,113^

The early studies on photoiniferter polymerization used UV light to activate iniferters, targeting the π–π* transition for trithiocarbonates and the n−π* transition for xanthates. Eventually, visible light polymerizations utilizing n−π* transition for trithiocarbonates and dithiobenzoates were reported.^8,9^ Comparison of methyl acrylate polymerization kinetics under blue and UV light revealed an increased induction period under blue light, after which both reactions proceeded at similar rates, though the light sources used in the study had different power outputs, resulting in different light intensities.^9^ Recent studies by Hughes et al. reported the excitation dependence of CTA photolysis and photoiniferter kinetics, with reaction rates varying based on the targeted electronic transition.^113^ For both xanthates and trithiocarbonates, targeting the symmetry forbidden n-π* transition led to increased C–S bond photolysis and initiation rates (Figure 6).In the case of a DMA (*N*,*N*-dimethylacrylamide) polymerization mediated with a trithiocarbonate, the apparent rate of polymerization was three times faster under blue than UV light with the same irradiance.^113^ Even a slight overlap of the RAFT agent absorption spectrum and light emission spectrum can be sufficient to successful photoiniferter polymerization.^114^ While xanthates are typically activated with UV light since their n−π* transition is around 350 nm, they are more photolytically active than both trithiocarbonates and dithiobenzoates, even at wavelengths >440 nm, which have minimal overlap with the xanthate absorption spectrum, as shown by electron spin trapping experiments.^115^ Nonetheless, when higher-energy light was used (λ > 390 nm), vinyl acetate polymerized faster because of the increased rate of photodissociation of the xanthate.

**Figure 6:**
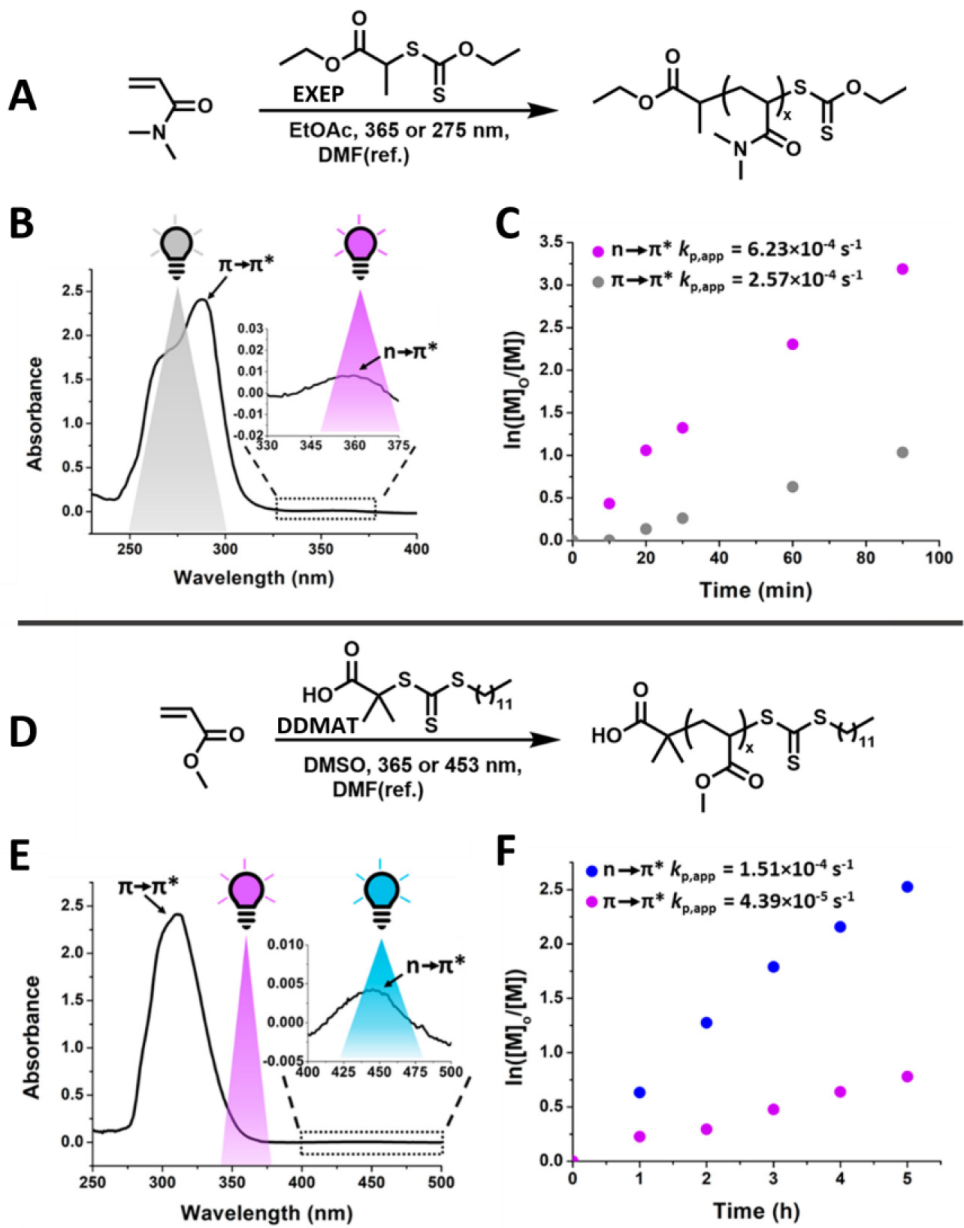
(A) Polymerization scheme of *N*,*N*-dimethylacrylamide with EXEP. (B) UV–vis spectrum of EXEP in ethyl acetate and the overlap of the absorbance with the wavelengths of light used. (C) Linear pseudo-first-order kinetic plot of the two xanthate-mediated photoiniferter polymerizations of DMA under different wavelengths of light. (D) Scheme for the photoiniferter polymerization of methyl acrylate with DDMAT under different wavelengths of light. (E) UV–vis spectrum of DDMAT in dimethyl sulfoxide and the overlap of the absorbance with the wavelengths of light used. (F) Linear pseudo-first-order kinetic plot of the two trithiocarbonate-mediated photoiniferter polymerizations of MA under different wavelengths of light. Adapted from ref 113. Copyright 2023 American Chemical Society.

Trithiocarbonates perform better under visible light, resulting in both faster and more controlled polymerization. Blue or green light is typically used for photoiniferter polymerizations within the visible range. In the seminal work on visible light photoiniferter polymerization, Boyer and co-workers found that only trithiocarbonate with a tertiary cyano R group (CDPA, 4-cyano-4-[(dodecylsulfanylthiocarbonyl)­sulfanyl]­pentanoic acid, Scheme 4) resulted in polymerization of MMA under green light while tertiary alkyl R group (DDMAT, 2-(Dodecylthiocarbonothioylthio)-2-methylpropionic acid) only worked under blue light, despite similar absorption in the green light region.^8^ This was attributed to the lower bond dissociation energy of CDPA than DDMAT. Furthermore, while CDPA could mediate the polymerization of methyl methacrylate under green light irradiation, the same reaction conditions led to no polymerization of methyl acrylate, likely due to the transformation of the effective R group from tertiary to secondary upon the addition of a single acrylate monomer unit to the RAFT agent. This is also consistent with the work reported on selective green light activation of CDPA in the presence of PABTC (2-(n-butyltrithiocarbonate)­propionic acid, Scheme 4), which was utilized to prepare bottlebrushes. A PABTC-functionalized methacrylic backbone was synthesized under green light (520 nm, 4.25 mW/cm^2^) followed by grafting of methyl acrylate monomer under blue light (465 nm, 6.5 mW/cm^2^).^95^ The lack of photolysis of PABTC under green light was attributed to the poor overlap of PABTC with green light. While PABTC can still participate in addition–fragmentation, the rate is suppressed because even if methacrylic radicals add to PABTC, methacrylate fragments preferentially due to increased radical stability. It was later shown that trithiocarbonates with a secondary R group could also be activated under green light, and well-defined PMA was achieved with a methyl ester equivalent of PABTC (PMBTC), provided that sufficient irradiance was used (102 mW/cm^2^at 527 nm light).^116^

**Scheme 4:**
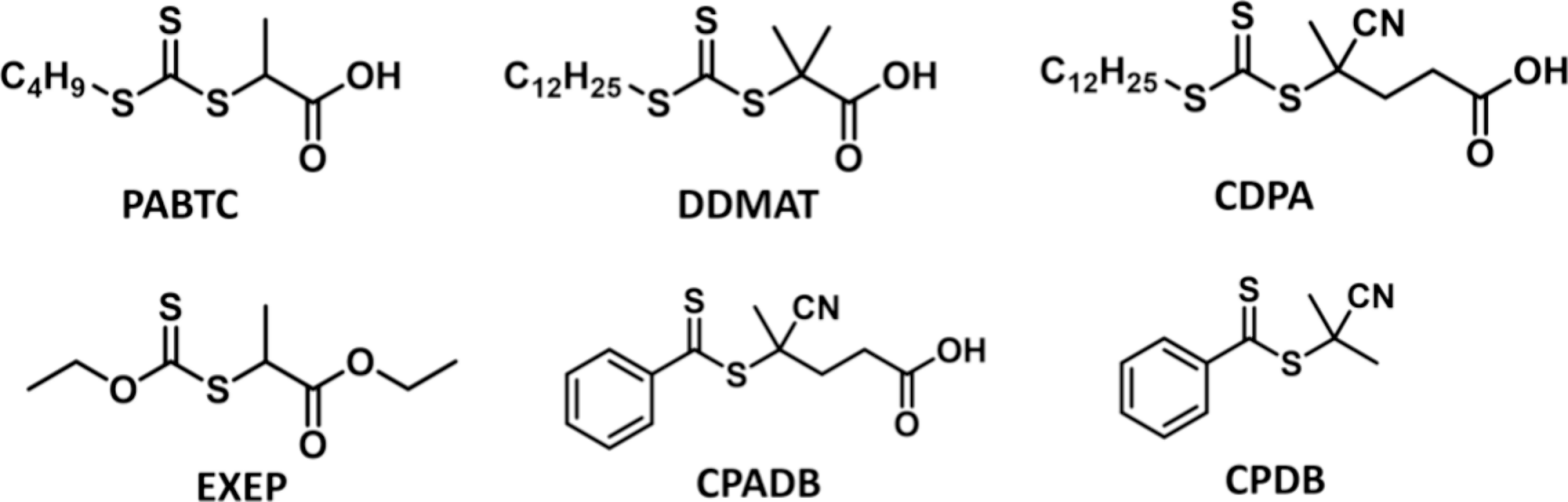
Structures of Commonly Used RAFT Agents in Photopolymerization

This hints at the role of the rate of radical recombination on the initiation and hence the success of polymerization. Studies under blue light showed that while both secondary and tertiary R group trithiocarbonates undergo photolysis, the secondary R group CTAs recombine much faster, thus lowering the apparent rate of photolysis.^117^ If photolysis rates are reduced under green light compared to blue light, statistically, there is a lower probability of a reaction of the R-group radical with the monomer, which will be more noticeable for CTAs with secondary R groups with higher recombination rates. Because tertiary R-group radicals are more sterically hindered, their recombination rate is lower, and hence,they are more likely to initiate polymerization. This is consistent with the lower rate of polymerization observed for photoiniferter polymerizations under green light compared to blue light. While the photodissociation of the RAFT agent is crucial for the success of polymerization, the radical stability of the monomer also contributes to the dilemma of wavelength choice. If the RAFT agent fragments recombine too fast after photolysis or the generated radicals have low reactivity toward the monomer, polymerization will not proceed.^118^

If blue light works for acrylic and methacrylic monomers and RAFT agents with secondary and tertiary R groups, does polymerizing methacrylates under lower energy green light bring any advantages? Blue light, as a more activating wavelength than green light, leads to increased radical concentration, which typically equates to a faster rate of polymerization. However, increased photolysis of macroCTA leads to increased radical concentrations, which can lead to more degradation of the polymer end groups. Indeed, for the polymerization of MMA, faster polymerization under blue light was associated with a concurrent reduction in control over polymerization, manifested by broader molecular weight distribution and discrepancies between experimental and theoretical weights.^95,116^ In summary, blue light typically leads to faster polymerization than green light; however, the latter gives better control over polymerization of monomers giving more stabilized propagating radicals.

### 5.3 Photostability of CTAs and macroCTA

Loss of control at higher energy wavelengths can be attributed to increased photolysis and degradation rates of the thiocarbonylthio group. In the early days of RAFT polymerization, photopolymerizations were carried out under UV light irradiation, but significant degradation of polymer thiocarbonylthio end groups was observed, especially at high monomer conversions and long irradiation times.^10,119^ Cutting off short-wavelength higher energy UV light with the aid of filters led to improved results by partially suppressing this degradation; however, the levels of control observed were still inferior to conventional thermal RAFT polymerization.^120,121^ UV light excites trithiocarbonates into a higher-energy S2 state, which increased lifetime increases the likelihood of degradation. Within the visible spectrum, despite all wavelengths promoting excitation to the S1 state, photodegradation rates increase with higher-energy light. The fastest photodegradation was observed under blue light (470 nm), followed by cyan (505 nm) and green light (527 nm) for a similar photon flux.^116^ This is likely a result of an increased rate of photolysis under blue light, which brings more opportunities for the thiocarbonylthio radical to degrade to carbon disulfide and the corresponding thiol radical. The extent of photodegradation can be followed with UV–vis spectroscopy by looking at the intensity of the characteristics π–π* absorption peak. Decomposition of the RAFT agent is more pronounced when higher degrees of polymerization are targeted as a higher fraction of CTA is degraded given the same irradiance and reaction time.^90^ Bai et al. followed the degradation of a trithiocarbonate under UV light with *in situ* NMR and found that a higher fraction of RAFT end group was retained at higher CTA concentration even under high irradiance­(48 mW/cm^2^) and broad range UV light.^122^

While the wavelength of light and irradiance can be adjusted if degradation is observed, the rate of irreversible decomposition of the RAFT agent is linked to its inherent photolytic stability. While no degradation was observed for trithiocarbonates with primary and secondary R groups under blue light (1.5 mW/cm^2^) irradiation for 15 h, up to 12% degradation was observed for the tertiary R group, with cyano-substituted R group having a higher rate of decomposition compared to alkyl-substituted R group (Figure 7).^117^ Dithioesters are more sensitive to light than trithiocarbonates or xanthates and are rarely used in photopolymerizations for this reason.^120^ As monomer adds to the RAFT agent and the nature of the R group changes, so will the photostability of the macroCTA. Monomers with tertiary propagating radicals, such as methacrylates and methacrylamides, are expected to lead to increased photodegradation of the end groups compared to secondary monomers, such as acrylates and acrylamides. Indeed, when the photostability of PMMA and PMA was studied in the absence of monomer, degradation was observed for PMMA and not for PMA, even at high irradiance (460 nm, 115 mW/cm^2^).^116^ For PMMA, complete degradation of the ω-end was observed within 2 h of blue light irradiation. The degradation rate decreased under green light (527 nm, 102 mW/cm^2^) compared to blue light.

**Figure 7:**
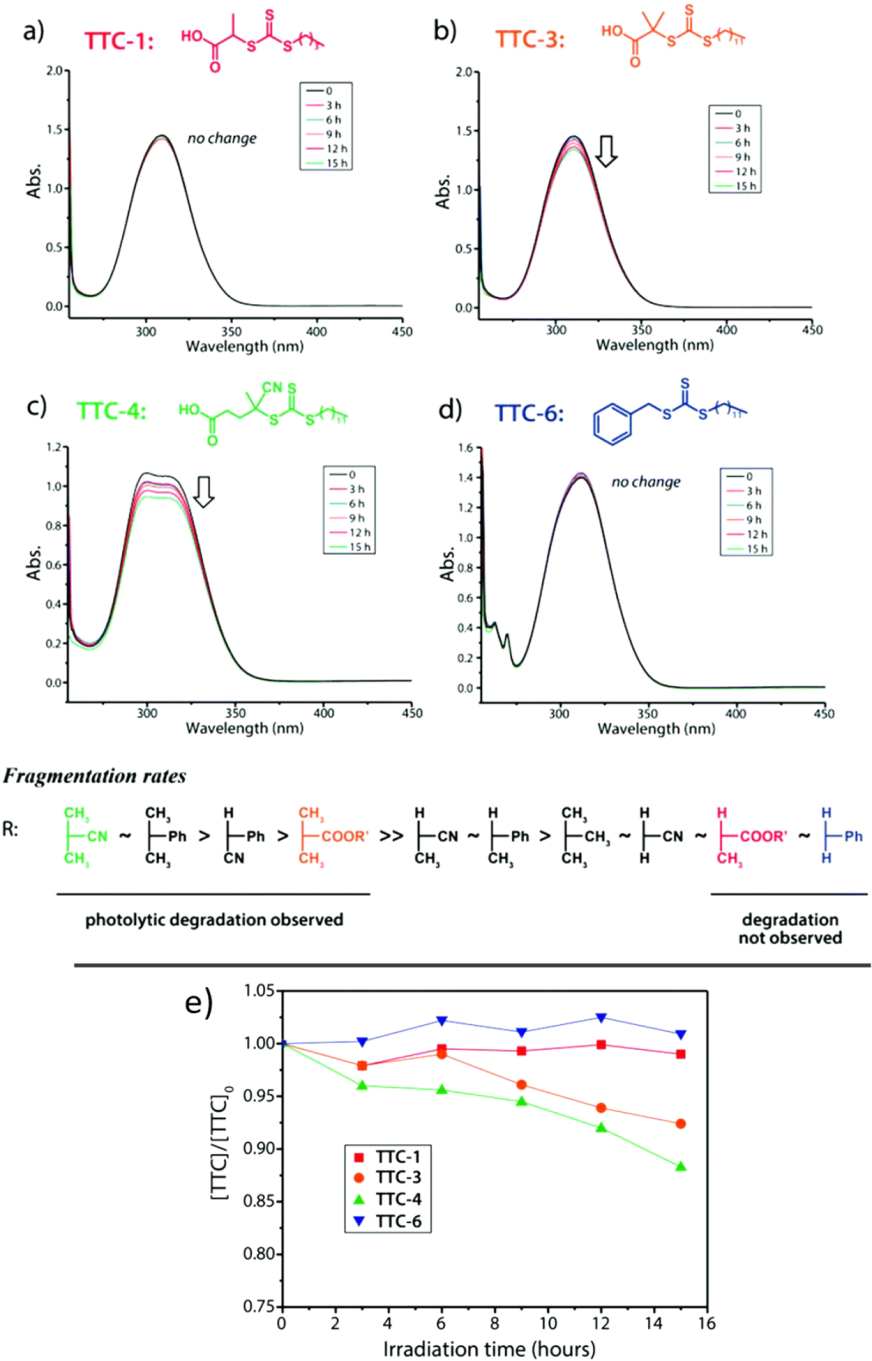
(a–d) UV–vis investigation into TTC-degradation in 55 mM DMSO solutions (deoxygenated) under blue LED irradiation (ca. 1.5 mW cm^–2^). (e) Degradation of various TTCs as determined via UV–vis spectroscopy using a peak absorbance value of 310 nm. Adapted from ref 117 with permission from the Royal Society of Chemistry. Copyright 2016 Royal Society of Chemistry.

The photostability of trithiocarbonates can be increased in the presence of a tertiary amine electron donor. Tertiary amines are able to transfer an electron to trithiocarbonates to generate a thiocarbonylthio radical anion and an amine radical cation­(Scheme 5).^123^ The TTC radical anion can then fragment to release a carbon-centered radical and a resonance-stabilized trithiocarbonate anion, which is suspected to be stable and less prone to decomposition to carbon disulfide and a Z-group radical. Significant improvement in control over polymerization of both hydrophobic and hydrophilic methacrylic monomers under UV irradiation (λ_max_ = 365 nm) was reported with *Đ* ≤ 1.30 at quantitative monomer conversions when 5 equiv of Me_6_TREN (tris­[2-(dimethylamino)­ethyl]­amine) with respect toCTA were added.^123^ The nature of the tertiary amine (TEA (triethylamine), PMDETA (*N*,*N*,*N*′,*N*″,*N*″-pentamethyldiethylenetriamine), Me_6_TREN) did not have a significant effect on kinetics and control of polymerization. No difference was observed when tertiary amine was added to polymerizations of *N*-isopropylacrylamide (NIPAM) and MA. Good control was achieved for both control and amine-catalyzed reactions. Yet, for amine-assisted photoiniferter polymerization of methyl acrylate under visible light, poor control was observed (*Đ* = 1.59), and for polymerization of vinyl acetate with a xanthate, addition of an amine resulted in a sluggish polymerization (7% monomer conversion in 30 h).^124^ Similarly, when TEA was added to xanthate-mediated polymerization of 4-acryloylmorpholine (NAM) under UV light, increased dispersities and decreased conversions were obtained.^66^ However, similar effects were observed for control thermal reactions, suggesting interference of the amine with the RAFT mechanism or partial hydrolysis of the CTA.

**Scheme 5:**
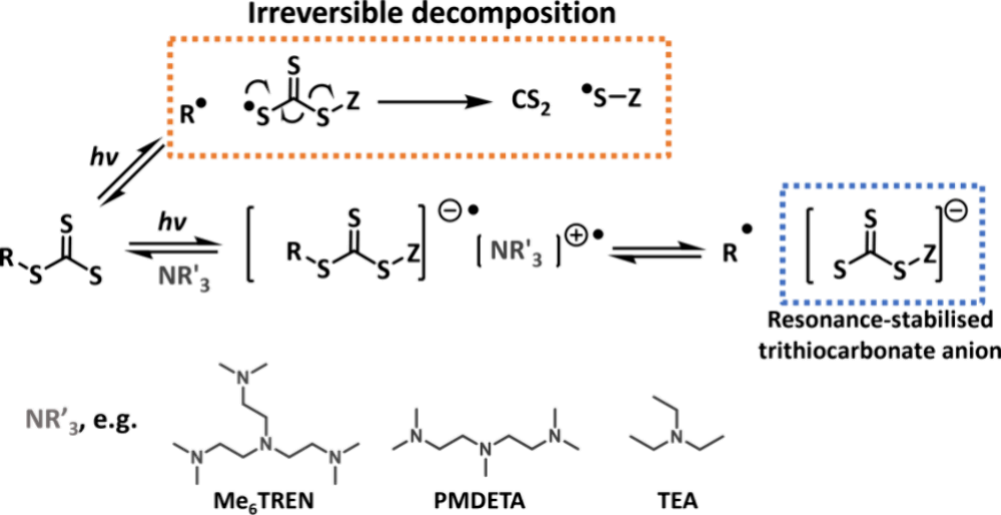
Decomposition of Trithiocarbonate RAFT Agent upon Excitation with Light and Amine-Assisted Formation of Stable Trithiocarbonate Anion

If photodegradation is linked to the inherent stability of the propagating chain end and the RAFT agent, how can it be prevented? This is where the optimization of reaction conditions becomes essential. Photodegradation studies in the absence of a monomer do not give a complete picture of the polymerization mechanism. Photodegradation of the RAFT agent can only happen during the pre-equilibrium stage of RAFT polymerization, so fast initialization and minimization of the induction period are prerequisites for control in photoiniferter polymerization. Similarly, decomposition of the thiocarbonylthio polymer end group occurs at a relatively lower rate in the presence of a monomer during polymerization. However, after complete monomer consumption, there is no further polymer growth possible, while chain transfer end degradation can continue at essentially the same rate; therefore, stopping the polymerization before complete monomer conversion and avoiding extensive irradiation with light (i.e., after complete monomer consumption) are essential.

## 6 Optimizing RAFT Photopolymerization—Kinetics, Control, and End-Group Fidelity

### 6.1 Effect of Catalyst, Additives, and Solvent

In PET-RAFT, the efficacy of the photocatalyst will inevitably affect the kinetics of the polymerization. Comparison of DMA polymerization catalyzed with Ir­(ppy)_3_ and Ru­(bpy)_3_Cl_2_ under blue light (435 nm) showed increased polymerization rate for the iridium catalyst, likely due to its higher redox potential and longer excited-state lifetime.^101^ The polymerization rate can be further modulated by altering the catalyst concentration. At higher catalyst loadings (up to a certain limit), decrease in the induction period and an increase in the apparent rate of polymerization are observed without a significant difference in polymerization control­(Figure 8).^102,125^ Eventually, at higher catalytic loadings, the rate enhancement plateaus as the system either becomes saturated and all photons are absorbed^125^ or self-quenching occurs at higher catalyst concentrations.^78^ Additionally, some catalysts, in particular porphyrins, due to their extended π-conjugated aromatic domains, can aggregate at higher concentrations due to π–π interactions, leading to quenching of photocatalyst.^126^ This can lead to slower polymerization kinetics at higher catalyst loadings.^127^

**Figure 8:**
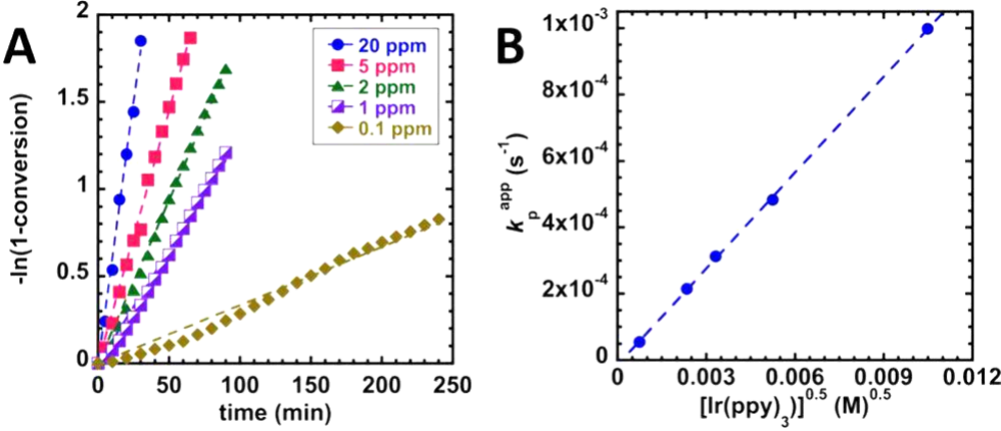
Kinetic data of MA polymerization with PABTC as the chain transfer agent and Ir­(ppy)_3_ as the catalyst under blue irradiation (460 ± 10 nm, 0.77 mW/cm^2^) in DMSO, [MA]_0_ = 5.5 M, at ambient temperature. (A) Semilogarithmic plot (dashed line is the line of best fit for a data set). (B) Scaling relationship for the apparent propagation rate (*k*_p_^app^) with the square root of [Ir­(ppy)_3_] (*R* ^2^ = 0.999). Reproduced from ref 125. Copyright 2018 American Chemical Society.

For photoiniferter polymerization, the addition of a tertiary amine was found not only to improve the photostability of the RAFT agent and end group but also to increase the rate of polymerization. For the polymerization of methacrylic monomers with dithiobenzoates under 440 nm light, an increase in polymerization rate was observed as the amine concentration was increased up to 6 equiv of amine with respect to CTA, after which a further increase in amine loading did not lead to significant rate enhancement; good control was observed in all cases (Figure 9).^124^

**Figure 9:**
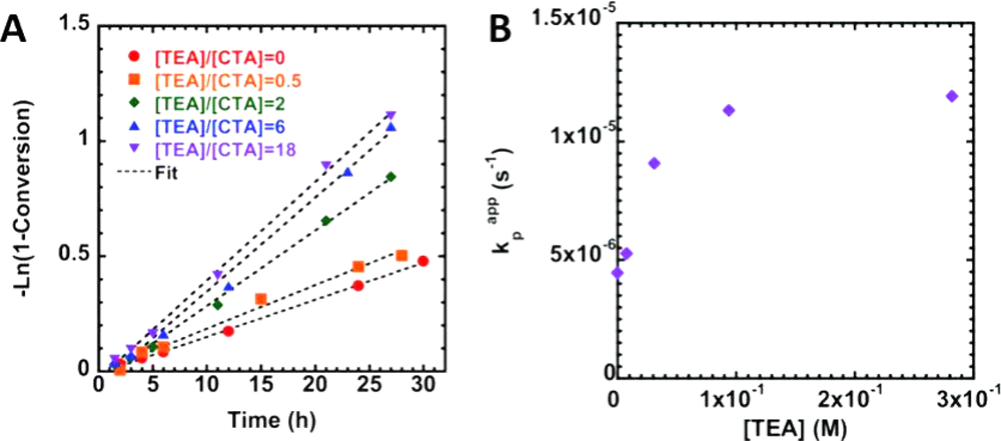
(A) First order kinetic plot, (B) dependence of the apparent rate of propagation (*k*_p_^app^) on the [TEA] for the polymerization of MMA using CPDB as the CTA in the presence of triethylamine (TEA) under blue light (λ_max_ = 440 nm, 11.6 ± 0.3 mW cm^–2^) with different TEA concentrations. Reproduced from ref 124 with permission from the Royal Society of Chemistry. Copyright 2016 Royal Society of Chemistry.

More recently, Antonopoulou et al. demonstrated that the addition of small quantities of citric acid to photoiniferter polymerization of water-soluble monomers (acrylamides, acrylates and methacrylates), resulted in a significant enhancement of polymerization kinetics and control,^128^ analogous to the observed effects in conventional RAFT polymerization.^129,130^ With increasing acid concentration, complete and rapid consumption of CTA was observed, accompanied by a gradual reduction and eventual elimination of the induction period at pH 3. Furthermore, the addition of acid resulted in reaction rates that were up to 3.4 times faster reaction rates. This methodology enabled the synthesis of well-defined high molecular weight polymers (DP = 3,000) within short time scales. The effect of acid was attributed to improved photolysis, likely arising from a decreased bond dissociation energy and modified excited-state energy levels of the protonated thiocarbonylthio compound.

Amine-assisted polymerization was shown to proceed faster in more polar solvents. Out of DMSO, dioxane, and toluene, polymerization proceeded fastest in DMSO while reducing solvent polarity led to a slower rate of polymerization and increased length of induction period with no significant differences in the molecular weight distributions of the resulting PMMA.^123^ Similarly, green light photoiniferter polymerization of MMA in the absence of amine proceeded faster in DMSO than in dioxane or toluene.^8^ PET-RAFT polymerizations are typically run in polar solvents to ensure good solubility of the photocatalyst, and more polar DMSO leads to faster polymerization than DMF.^125^

DMSO leads to faster polymerization kinetics and increased oxygen tolerance, its high boiling temperature makes it difficult to remove after reaction, and hence it is not suitable for many applications.^131^ Similarly, deep eutectic solvents (DES) were also shown to increase the rate and control of both photoiniferter and PET-RAFT polymerization; however, such solvents also have high boiling point. Photoiniferter polymerization of MMA in hydrophobic DES–a tetrabutyl-ammonium chloride and ethylene glycol mixture was found to proceed effectively in an open-to-air system; albeit a longer induction period was observed compared to the DMSO system, overall, 2.8 and 4.5 fold rate enhancement were observed for trithiocarbonate and dithiobenzoates RAFT agents, respectively. The effect was attributed to the formation of monomer domains within the DES leading to increased local concentration of radicals and monomer with a simultaneous reduction in the rate of termination.^132^ PET RAFT in DES using EY as a photocatalyst showed a lower rate increase but demonstrated oxygen tolerance when the reaction was carried out in the open air.

An ionic liquid (IL) is a salt that is liquid at room temperature and is composed of organic cations and inorganic anions. ILs possess properties such as a low melting point, low vapor pressure, high thermal stability, and high ionic conductivity. When photoiniferter polymerization was conducted in IL/water as the solvent using 50–100% 1-ethyl-3-methylimidazoliumethyl sulfate [EMIM]­[EtSO_4_] with hydrophilic monomers such as DMA, a significant increase (7-fold) in the polymerization rate was observed.^133^ Additionally, when photoiniferter polymerization under blue light was performed with hydrophobic monomers in a 50% IL ([EMIM]­[PF_6_]) and DMSO solvent mixture, a 3.3-fold rate increase was observed compared to pure DMSO.^134^ This system exhibited both temporal control and oxygen tolerance. In both cases, the IL was successfully recycled.

The [EMIM]­[EtSO_4_] IL was also used as a (co)­solvent for PET-RAFT polymerization of DMA with varying levels of EY as an organic photocatalyst under blue and green light irradiation. It was observed that partial or full substitution of the reaction solvent with IL accelerated the PET-RAFT rate without requiring any cocatalyst.^135^ Oxygen tolerance was again observed, and under both blue and green light, only a very low concentration of EY was required.

Although typically photopolymerizations are carried in homogeneous media that provide excellent light penetration,^136^ successful miniemulsion conditions were reported for both PET-RAFT^137^ and photoiniferter polymerizations.^69,138–140^ Sumerlin and co-workers demonstrated that the synthesis of ultrahigh molecular weight polymers can be successfully adapted to miniemulsion conditions.^69,138,139^ This modification significantly enhances the processability of these polymers by reducing the overall viscosity while preserving a high viscosity within the reaction droplets, which is essential for minimizing termination events. Compartmentalization of the reaction, occurring within dispersed systems, can further enhance reaction rates and provide greater control over the polymerization.^141,142^

### 6.2 Light Intensity and Reaction Temperature

When describing reaction conditions, often *light intensity* is incorrectly used in place of *irradiance*.^94^ While light intensity describes the power emitted by a light source, irradiance describes how much power is received by a surface (e.g., reaction vessel), expressed in watts per square meter (W/m^2^, SI unit). Intensity can be used to describe the source of light; however, in the context of photochemical reactions, the irradiance is more valuable measure as it describes the incident power per unit area.

Increased light intensity means that more photons are being emitted. As more photons bombard the photoactive compound, the rate of photoexcitation and the frequency of chemical events resulting from the excited state chemistry increase. For both PET-RAFT and photoiniferter polymerization, this leads to an increased rate of fragmentation of (macro)­CTA, albeit the relationship between light intensity and photolysis will differ for both mechanisms as the light-absorbing species is different in those systems.

For a typical PET-RAFT polymerization (polymerization of MA with and Ir­(ppy)_3_ as a catalyst in DMSO under blue light (460 nm)), the apparent rate of polymerization scaled with 1/2 order of light intensity (Figure 10).^125^ For polymerizations operated without exclusion of oxygen, light intensity was found to significantly impact the length of the induction period—at lower irradiance values, longer induction periods were apparent as slower generation of radicals results in slower consumption of oxygen.^102^ Importantly, increased light intensity did not have a detrimental impact on control over polymerizations, though the irradiance values employed were low (<12 mW/cm^2^).

**Figure 10:**
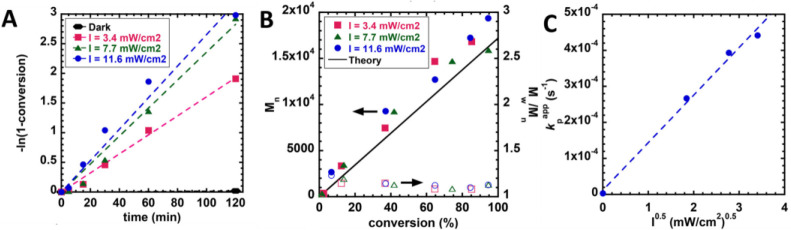
(A) Semilogarithmic plot (dashed line is the line of best fit for a data set) and (B) evolution of Mn (solid points) and *M*_w_/*M*_n_ (open points) of PET-RAFT polymerization under different light intensities of blue (440 ± 10 nm) radiation. (C) Scaling relationship for the apparent propagation rate (*k*_p_^app^) with the reciprocal square root of the photoreactor intensity (*R* ^2^ = 0.991). Reaction conditions [MA]_0_:[PADTC]_0_:[Ir­(ppy)_3_]_0_ = 200:1:0.001, [MA]_0_ = 4.9 M, in DMSO:DMF = 7:3 at ambient temperature. Adapted from ref 125. Copyright 2018 American Chemical Society.

In photoiniferter polymerization, an increase in the rate of (macro)­CTA photolysis will also result in an increased rate of reversible deactivation, limiting the effective radical concentration. Indeed, the rate of photoiniferter polymerization has been reported to increase linearly with light intensity until a plateau is reached, and a further increase in light intensity does not result in an increase in the rate of polymerization.^143^ In a study by Ammini et al., irradiance values greater than 7.95 mW/cm^2^ did not result in a further increase in polymerization rate, an observation ascribed to saturation of the system with light (Figure 11);^144^ however, other studies reported enhancement for different photoiniferter polymerizations when irradiance was increased beyond this value.^118,122,145^ The exact value of the maximum cutoff irradiance is likely affected by monomer type, degree of polymerization, and reaction conditions such as temperature. For fast propagating monomers and/or at elevated temperatures that promote propagation, the threshold irradiance above which no further changes to polymerization rates are observed will be likely higher than for slow propagating monomers, such as methacrylates for which propagation may become the rate-limiting step rather than photodissociation.^118,144,146^ However, both acrylic and methacrylic monomers were reported to have a linear relationship between polymerization rate and light intensity, as the rates of both photolysis and reversible deactivation depend on the stability of generated radicals (Figure 11).^144,146^

**Figure 11:**
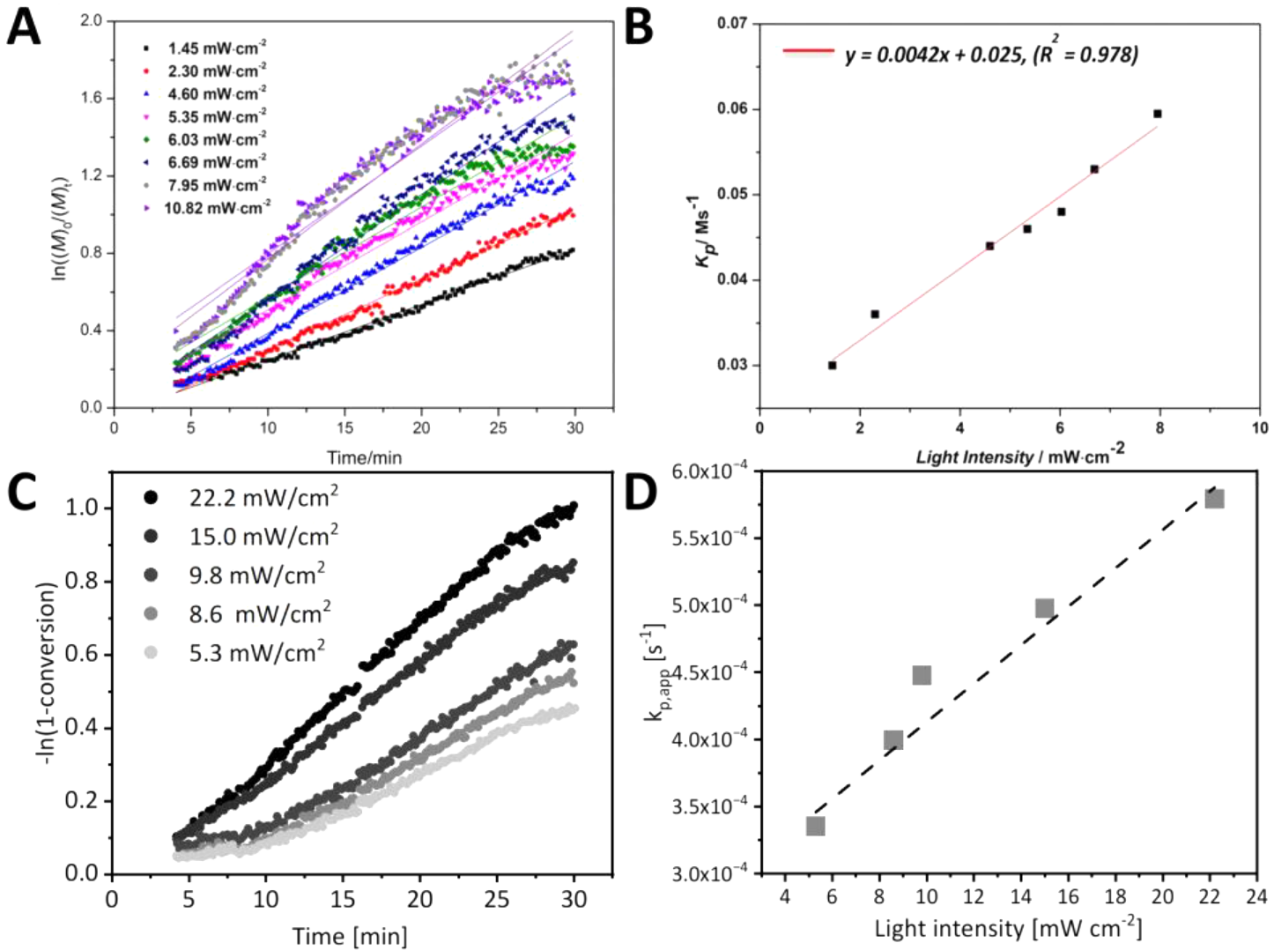
(A) Pseudo-first-order kinetic plot and (B) scaling relationship for apparent propagation rate with light intensity for MMA photoiniferter polymerization with CDPA under blue light (450 nm) with [MMA]/[CDP-TTC] = 25, [MMA] = 3 M, at 90 °C in DMSO. (C) Pseudo-first-order kinetics plot and (D) scaling relationship for apparent propagation rate with light intensity for MA photoiniferter polymerization with PABTC under blue light (460 nm) with­[MA]/[PABTC] = 50, [MA] = 3 M at 70 °C in DMSO. (A, B) Reproduced from ref 144 with permission from the Royal Society of Chemistry. (C, D) Reproduced from ref .^146^ with permission from the Royal Society of Chemistry. Copyright 2024 Royal Society of Chemistry.

While the increased reaction rates at higher light intensities are well documented, studies are limited to low to moderate irradiance values, and the effect of high irradiance values on polymerization control is not well documented. One study looked at UV light irradiance ranging from 3 to 48 mW/cm^2^ for photoiniferter polymerization of MA and found minimal broadening of molecular weight distribution; however, reactions were only taken to 20% monomer conversion.^122^ Another study on photoiniferter polymerization found a significant decrease in the dispersity of PNIPAM (1.28 to 1.05 for similar molecular weights achieved) as the distance of the light source from the reaction vessel was increased, resulting in lower irradiance at the reaction vessel.^147^ For photoRAFT polymerization with a photoinitiator at 365 nm, a gradual loss of control was observed as irradiance was increased from 5 to 70 mW/cm^2^.^148^ As RAFT agents also absorb light in the UV region, the loss of control can be attributed to increased participation of photoiniferter activation pathway at increased light intensities and competing reaction mechanisms.

While further research is necessary, diminished control is generally anticipated at high irradiance due to increased photodegradation and increased termination resulting from higher radical concentration, just like in conventional thermal RAFT polymerization. Alternatively, the rate of polymerization can be increased by elevating the reaction temperature. In photopolymerization, the temperature should not significantly affect initiation or termination but will substantially impact the propagation rate. Lewis et al. showed that by using high-power blue light and a concomitant increase in reaction temperature to 80 °C, the reaction time to achieve high acrylamide conversion (>85%) could be shortened from 12 h to just 5–11 min.^145^ Junkers and co-workers showed significant acceleration photoiniferter polymerization of isoprene under UV irradiation at elevated temperatures, with an increase in conversion from 45 to 78% being observed as the temperature was raised from120 to 145 °C.^149^

While photoiniferter polymerization can operate at elevated temperatures, care must be taken for photopolymerizations involving photoredox catalysts, as some of these compounds are sensitive to temperature. One of the examples includes the ruthenium-based catalyst Ru­(bpy)_3_Cl_2_, which is commonly used in PET-RAFT. Even at moderately increased temperatures, this catalyst can be efficiently quenched due to the thermal population of metal-centered excited states.^150^ For RAFT polymerization with a benzaldehyde photoredox catalyst, a 5-fold rate acceleration was observed when the reaction temperature was increased from 33 to 50 °C without adverse effects on reaction control.^151^ Similarly, a rate enhancement was reported for photoiniferter polymerization of methacrylates at 90 °C.^152^ The combination of elevated temperature and flow chemistry significantly reduced the reaction time, which limited the degradation of trithiocarbonate. As RAFT agents have high thermal stability, increasing the reaction temperature is an excellent method for shortening the reaction time and improving control over polymerization.

### 6.3 Different Target Molecular Weights

Both photoiniferter and PET-RAFT polymerization methods can be employed to target polymers across a broad range of molecular weights. However, in practice, the photoiniferter polymerization of high molecular weight polymers proceeds slowly, whereas PET-RAFT polymerization of low molecular weight polymers can be retarded, like many thermal RAFT polymerizations.^52,153^ Significant rate retardation and increased induction periods were observed for PET-RAFT with a target degree of polymerization DP = 50 compared to DP = 400, with the apparent rate of polymerization scaling with [CTA]^−1/4^.^102^ This suggests that excitation of the photocatalyst is a rate-determining step for radical generation in PET-RAFT. At lower target degrees of polymerization, the concentration of CTA is higher, leading to more competition between CTA and photocatalyst for photon absorption, especially at UV and blue light wavelengths, which can be overcome by the use of low energy of wavelengths (red or near infrared lights). As the monomer concentration was increased from 1.9 to 4.9 M and the absolute CTA concentration was increased at a fixed target degree of polymerization, a minimal rise in polymerization rate was observed despite an increased rate of propagation at higher monomer concentration, highlighting the importance of optical transparency which is decreased at higher CTA concentration.^125^ Contrary, in photoiniferter polymerization increased CTA concentration results in an increased rate of polymerization.^37^ In photoiniferter polymerization, the CTA acts as a chromophore, and the radical concentration is directly linked to the rate of photolysis and the concentration of the RAFT agent. This equates to slower polymerization rates and longer reaction times required for polymerizations targeting higher molecular weights.^144^ Yet, despite overall slower kinetics and increased degradation at low concentration of CTA, when photoiniferter polymerization is well optimized, even well-defined ultrahigh molecular weight polymers can be prepared. This was demonstrated for both homogenous aqueous^38,154^ and organic systems,^37,155,156^ as well as in heterogeneous aqueous mediasystem^37,69,138,139^ but polymerizations of hydrophobic (acrylic and styrenic) monomers require higher monomer concentrations (4 M compared to 2 M for aqueous systems) to maintain excellent control and fast reaction kinetics. Further, monomer feeding can improve the preparation of higher molecular weight polymers. Reducing the number of monomer molecules per growing chain at any given time increases the number of activation/deactivation cycles per monomer addition, leading to narrower molecular weight distributions.^66^ However, if monomer feeding is too slow, diminished control is observed, as in the absence of a monomer, end-group degradation is more prevalent.

## 7 Monomer Scope and RAFT Agent Selection

So far, we have discussed how different monomers require specific wavelengths of light, especially for photoiniferter polymerization (section 5), the selectivity of some PET-RAFT catalysts (section 3.3.1), as well as other factors affecting rate and control over polymerization (section 6).Furthermore, in section 2, we showed how the reactivity of the monomers dictates RAFT agent choice in conventional RAFT polymerization to ensure fast initiation of polymerization and efficient degenerative chain transfer. While the reactivity match is also crucial for light-mediated polymerization, subtle differences need to be considered in the context of photoactivation.

As the R group has a significant effect on the photolysis of the RAFT agent, differences in initiation are expected across different RAFT agents in photoiniferter polymerization. Trithiocarbonates with secondary R groups were shown to give an induction period in polymerization of methyl acrylate under blue light, whilefor well-fragmenting tertiary R groups, this was not observed (Figure 12A).^117^ Dithiobenzoates could not successfully initiate polymerization of methyl acrylate, regardless of the R group, and this was attributed to a significant change in bond dissociation energy upon the addition of one acrylate monomer unit to the dithiobenzoate, which halted photodissociation of the adduct. However, even in thermal polymerization, dithiobenzoates lead to significant inhibition (several hours) of the polymerization of methyl acrylate.^51^ Typically, trithiocarbonates with propionic acid R group (Figure 12A TTC-1 and TTC-2) exhibit excellent control over the polymerization of acrylates, and no induction or retardation is observed in conventional thermal RAFT polymerization as the R group radical is structurally similar to methyl acrylate propagating radical.^157,158^ The induction period observed for such RAFT agents in photoiniferter polymerization can therefore be attributed to the relatively slow rate of photolysis and the slow initiation of polymerization. When kinetics of methyl acrylate polymerization mediated with methyl 2-(((butylthio)­carbonothioyl)­thio)­propanoate (PMBTC) were studied across different light intensities, it was found that the induction period was only observed for low light intensities.^159^ When polymerizations of methyl acrylate were run at elevated temperatures (15 mW/cm^2^ and 70 °C), conditions favoring propagation, only tertiary and not secondary R groups (2–2-methylpropanoate and 2-cyanopropan-2-yl) resulted in an induction period, both for xanthates and trithiocarbonates, contrary to previously reported results (Figure 12). This highlights that some differences observed between conventional thermal RAFT and photopolymerization can result from lower temperatures rather than light activation. As xanthates photolyze very efficiently, even under blue light, this is likely a result of the increased time required to establish the RAFT equilibrium. More stable R-group radicals are also less reactive and have lower affinity to monomers, giving secondary propagating radicals.^159^

**Figure 12:**
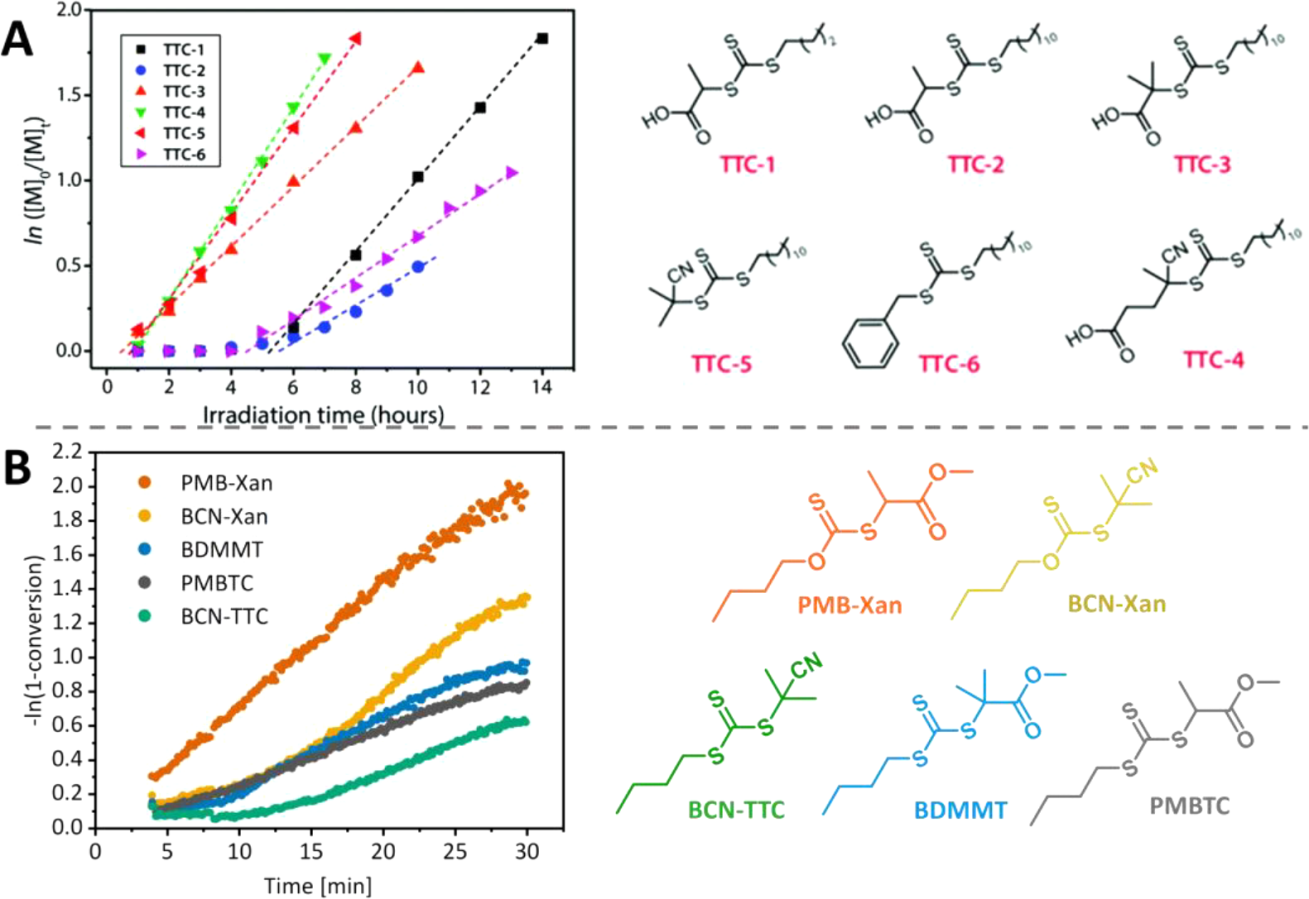
Photoiniferter photopolymerization of methyl acrylate using various RAFT agents. (A) Batch reaction, reaction conditions: [MA]:[CTA] = 100, [MA]_0_ = 3 M in DMSO, blue light (460 nm, ∼1.5 mW cm^–2^), ambient temperature. (B) Reaction in continuous flow, reaction conditions: [MA]/[CTA] = 50, [MA]_0_ = 3 M in DMSO, blue light (460 nm, 15 mW/cm^2^) at 70 °C. (A) Adapted from ref 117 with permission from the Royal Society of Chemistry. (B) Reprinted from ref 163 with permission from the Royal Society of Chemistry. Copyright 2014 Royal Society of Chemistry.

Different trends are observed for methacrylates. Methacrylates are notoriously more challenging to polymerize under light, both with photoiniferter and PET-RAFT polymerization. When PET-RAFT polymerization of methacrylates, acrylates, and acrylamides were compared under identical conditions but with different, suitable for each monomer family, RAFT agents, obvious diminished control and slower reaction progress was observed for methacrylates due to slower establishment of RAFT equilibrium and/or termination of growing polymer chains (Figure 13).^160^ Similarly, while well-defined ultrahigh molecular weight polyacrylates and polyacrylamides with number-average molecular weights over 1,000,000 g/mol could be prepared, lower-than-expected molecular weights and high dispersities were observed for MMA, which could be partially circumvented by addition of one molar equivalent of a tertiary amine, such as PMDETA.^37,68^

**Figure 13:**
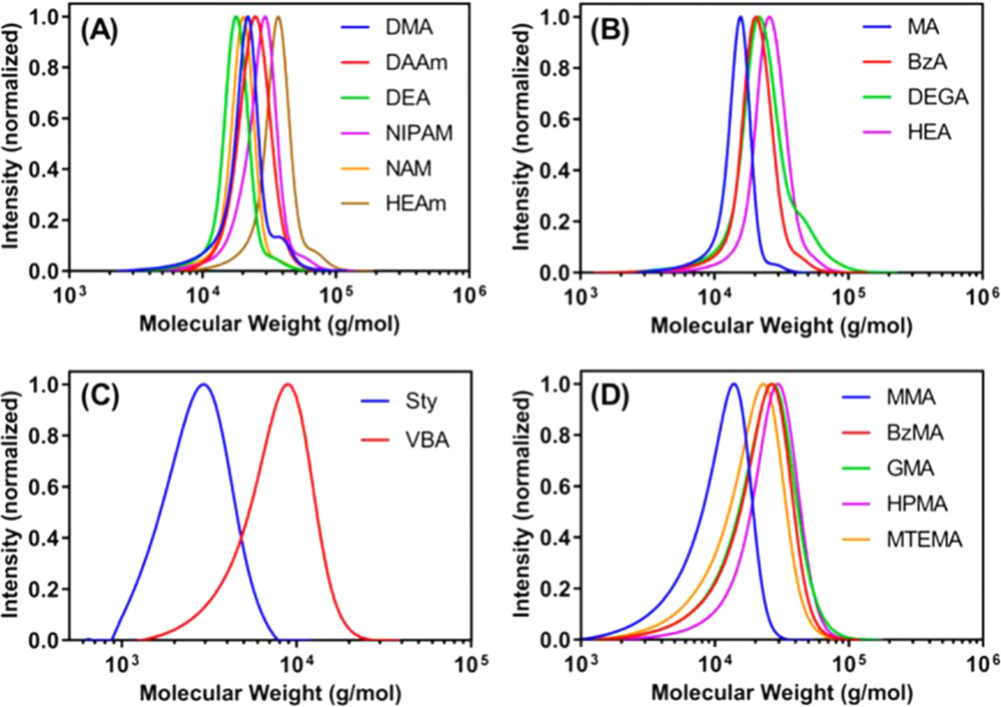
Molecular weight distributions of (A) acrylamide, (B) acrylate, (C) styrene, and (D) methacrylate monomer families prepared by using high-throughput PET-RAFT polymerization in 96-well plates. Polymerizations were conducted for 4 h under yellow LED light (560 nm, 9.7 mW/cm^2^), [M]:[CTA]:[ZnTPP] = 200:1:0.02, [M]_0_ = 1 M. Reprinted from ref 160. Copyright 2018 American Chemical Society.

In PET-RAFT, methacrylates are typically polymerized with a RAFT agent with a tertiary cyano R group, either dithiobenzoate (such as CPADB­(4-cyano-4-(phenylcarbonothioylthio)­pentanoic acid) or CPDB (2-cyanoisopropyl dithiobenzoate), suitable for most photocatalysts) or trithiocarbonate, such as CDPA, which works well with ZnTPP (Scheme 4). PET-RAFT polymerization of methacrylate monomers is significantly slower than that of acrylates or acrylamides. While monomer conversion above 80% can be achieved with 1 ppm of either Ru­(bpy)_3_Cl_2_ or Ir­(ppy)_3_ catalyst in 3 h for methyl acrylate and *N,N*-dimethyl acrylamide, methyl methacrylate required 36 h to reach similar conversions even with 5 ppm catalyst loading.^101^ While with ZnTPP, 90% monomer conversion was achieved for MMA in 12 h under red light irradiation, it required significantly higher catalyst loading − 50 ppm.^75^

All these problems can be attributed to previously mentioned differences in propagation, photodegradation, and termination rates. While both acrylates and methacrylates belong to the family of more-activated monomers, there are clear differences in their RAFT polymerization kinetic behavior.^52^ Photoiniferter polymerization of methyl methacrylate was almost completely inhibited by trithiocarbonates with secondary R groups.^144^ RAFT agents with less stable R groups get poorly incorporated on the growing polymer chains and as a consequence poor control over polymerization.^159^ Analogously to thermal RAFT, photopolymerization of methacrylic monomers can only be controlled with trithiocarbonates with tertiary cyano-substituted R groups and dithiobenzoates.^75,144^ For the latter, the effect of the substituent on the benzene ring was shown to have a remarkable impact on the rate of polymerization.^99^ As carbon–sulfur bond scission is associated with oxidation of the thiocarbonylthio group and reduction of the R group to a carbon-based radical, the electron density of the C=S bond will have a dramatic effect on the rate of CTA photolysis. Within dithiobenzoate RAFT agents, para substituents on the Z-group have a remarkable effect on bond dissociation—electron-donating groups significantly increase the rate of photolysis while electron-withdrawing groups decrease it despite similar UV–vis absorbance.^99^

While typically photoiniferter and PET-RAFT are applied to acrylates, methacrylates, and acrylamides, successful reports of methacrylamide,^143^ styrene, and isoprene^149^ polymerizations have also been reported. The main challenge for the latter is their slow rate of propagation, although this can be overcome with high reaction temperature and flow chemistry.

Among RDRP techniques, RAFT is most suited for the polymerization of less activated monomers^161^ and unconjugated monomers such as vinyl esters, and *N*-vinylpyrrolidinone can also be polymerized under light. Blue light PET-RAFT with Ir­(ppy)_3_ was shown to successfully control polymerization of vinyl acetate, vinyl pivalate, *N*-vinylpyrrolidinone, dimethyl vinylphosphonate, vinyl benzoate, and *N*-vinylcarbazol.^162^ Xanthate RAFT agent proved more successful than dithiocarbamates, likely due to more efficient activation of xanthate by the photocatalyst. However, polymerizations were relatively slow, and to achieve high monomer conversion, a 15–20 h reaction time was required, depending on the catalyst loading. Polymerization kinetics were also significantly affected by the solvent. Photoiniferter xanthate-mediated polymerization of vinyl acetate is notably faster than PET-RAFT, with less than 2 h required to achieve high monomer conversion,^115^ a period that could be further accelerated by increased reaction temperature.^163^

A general restriction in photoiniferter polymerization results from the duality of purposes of the CTA. While in a RAFT process, the RAFT agent has to be adjusted well to the monomer (possessing sufficient chain transfer capabilities to enable low dispersities), it also dictates the photochemical requirements (irradiation wavelength, intensities, etc.). These properties are not always well-matched, as for instances xanthates, which are extremely efficient iniferters have low chain transfer coefficients with most acrylic monomers. This issue can be addressed by mixing the CTAs. In the recently described xanthate-supported photoiniferter polymerization one RAFT agent (xanthate) is primarily responsible for the photoactivation and the other (trithiocarbonate) asserts control over the polymerization.^164^ Hence, dispersity can be controlled as a function of CTA ratios, and as both types of CTA are included as end groups in the ensemble of polymers, chain extension to form (multi)­block copolymers is easily possible.

## 8 Complex Polymer Architectures

One of the most powerful features of RAFT polymerization is the preparation of multiblocks,^165,166^ which is also a strong feature of photopolymerizations mediated with thiocarbonylthio compounds. For instance, well-defined pseudo hexablock polymers of NAM with minimal low molecular weight tailing were prepared in a high-throughput fashion with PET-RAFT without degassing and purification between monomer additions in a multiwell plate.^160^ Monomer conversion of at least 96% was achieved for each block. However, the reaction time had to be increased by an hour with every other chain extension to account for reduced reactant concentration and slower polymerization due to the dilution effect. Importantly, owing to the high photocatalytic efficiency of ZnTPP, no extra photocatalyst had to be added throughout the chain extension process and the initial loading of the catalyst was sufficient for both initiation and degassing of subsequent chain extensions. Pushing the limits even further, PET-RAFT can be used to synthesize multiblock star polymers with diminished star-to-star coupling compared to thermal RAFT.^167^ Stars with up to 10 arms with only a very gradual increase in dispersity with each block were prepared (Figure 14). Compared to thermal RAFT, improved control was ascribed to the absence of initiator-derived linear chains and diminished bimolecular termination.

**Figure 14:**
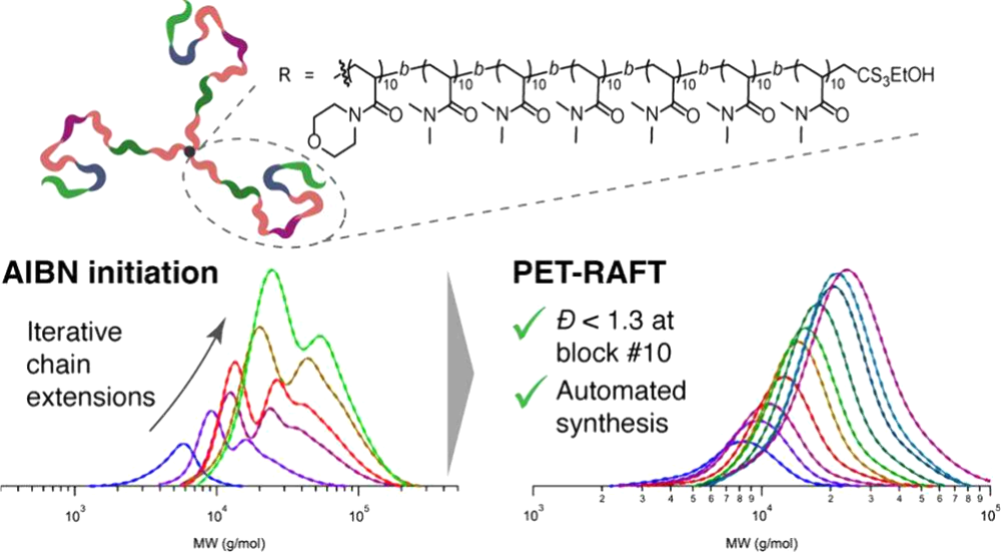
Comparison of multiblock star polymerizations of dimethylacrylamide by thermal and PET-RAFT polymerization methods. Reprinted from ref 167. Copyright 2022 American Chemical Society.

Photoiniferter polymerization can also be successfully used to prepare multiblock copolymers; however, with each monomer addition, the end-group concentration decreases, and the irradiation time necessary to achieve high monomer conversion inevitably increases. Icosa-block acrylamide copolymer (20-block) with block lengths from 20 to 100 repeat units was prepared with narrow dispersity (*Đ* = 1.29) within less than 52 h under UV light irradiation.^66^ Interestingly, the synthesis of a PNAM homoblock of DP 1000 led to a less well-defined polymer than a 20-block with cumulative DP 1000, likely due to the increased number of reversible deactivation steps per monomer addition for the multiblock. To expand the monomer scope of this approach, xanthate-supported photoiniferter polymerization can be used. While the number of blocks is more restricted (likely due to side reactions of the trithiocarbonate under UV light), livingness is drastically increased when compared to a photoiniferter polymerization using only a trithiocarbonate under these conditions.^164^

Iniferter polymerization can also be used to produce inverted block copolymers with acrylate or acrylamide block preceding methacrylate block with regular xanthate and dithiocarbamates RAFT agents owing to efficient reversible deactivation of corresponding macro chain transfer agents.^67^

Photoiniferter polymerization is also well suited for the preparation of star polymers due to diminished termination and the absence of initiator-derived polymers not attached to the core of the star. Ultrahigh molecular weight stars with 21 arms and 11 000 monomer units per arm were prepared using a core-first R-group approach.^39^ Typically, high molecular weight star polymers are difficult to prepare due to either steric hindrance in the Z-group approach (Z-group of the RAFT agent attached to the star core) or star–star coupling in the R-approach due to radicals formed at the ends of growing arms. Yet, diminished termination in photoiniferter polymerization led to the successful synthesis of well-defined star polymers with high end-group fidelity. Similarly, photoiniferter polymerization led to an improved result of bottlebrush polymer synthesis via both grafting-through and grafting-from approaches with no detectable bottlebrush–bottlebrush coupling, fewer linear chain impurities, and higher conversions, which were attributed to lower radical concentration, lack of initiator-derived chains and reduced chain coupling and β-scission due to lower reaction temperature.^36^

## 9 Oxygen Tolerance

One emerging advantage of photopolymerizations is benign methods of oxygen removal, such as photochemical deoxygenation.^168^ Typically, RDRP polymerizations require degassing, as oxygen can terminate growing chains. In photopolymerization, triplet oxygen can be photochemically converted to singlet oxygen or peroxide species, which can be scavenged by a quencher. In PET-RAFT processes, a photocatalyst in its excited triplet state can quickly quench triplet ground state oxygen into reactive oxygen species, including singlet oxygen and superoxide. Singlet oxygen can then be consumed by singlet oxygen scavengers such as DMSO; hence, PET-RAFT polymerizations are typically run in DMSO as a solvent.^169^ Different quenchers such as 9,10-dimethylanthracene or ascorbic acid can be added to achieve oxygen tolerant polymerization in different organic solvents^170^ and aqueous systems;^171^ this is particularly appealing as DMSO is a high boiling temperature solvent and as such is not suited for many applications. Consumption of oxygen at the beginning of the reaction by the photocatalyst leads to an induction period during which no polymerization takes place, with the notable exception of the ZnTPP catalyst, which did not result in any substantial increase in the length of the induction period.^75^ Once oxygen is consumed, polymerization starts. Depending on the reactivity of the photocatalyst and the extent of oxygen removal, polymerization can proceed with the same rate as degassed reaction, as reported for Ir­(ppy)_3_ catalyst,^53^ or if the oxygen removal is slow and incomplete, slower apparent rate of polymerization is observed due to reduced concentration of propagating radicals, as observed for Ru­(bpy)_3_Cl_2_ and ZnTPP (Figure 15).^75,101^ While PET-RAFT polymerization in the presence of oxygen results in a slight increase of polymer dispersity compared to a degassed system, molecular weight distributions remain narrow (*Đ* < 1.2).^172^

**Figure 15:**
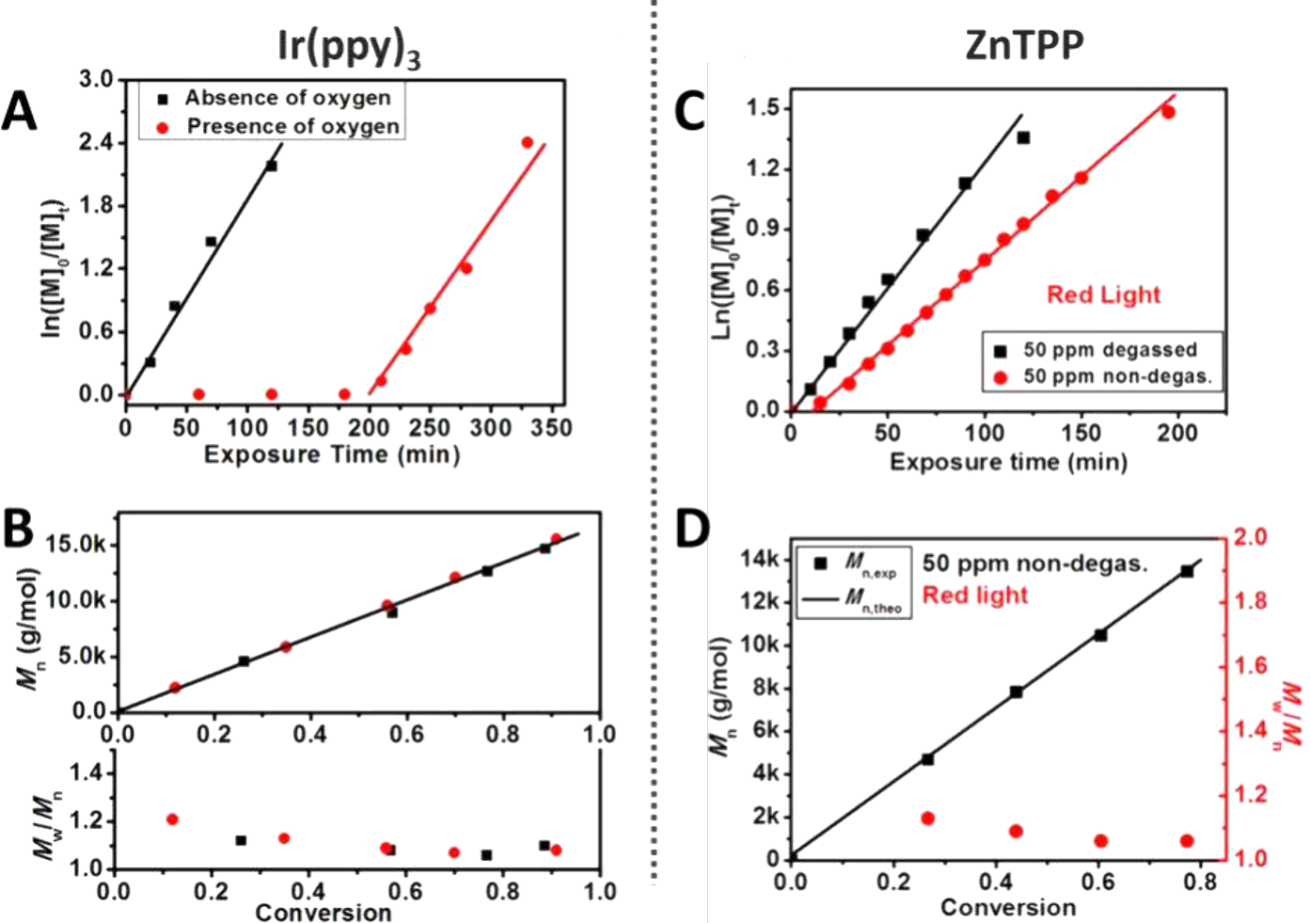
Kinetics plots for PET-RAFT polymerization of MA with Ir­(ppy)_3_ under blue light (435 nm) in DMSO (A, B) and ZnTPP under red light (635 nm) in DMSO (C, D) in the presence and absence of oxygen. (A, C) ln­([M]_0_/[M]_t_) versus exposure time for plots. (B, D) *M*_n_ and *M*_w_/*M*_n_ evolution with monomer conversion. Adapted from ref 53. Copyright 2014 American Chemical Society. Adapted from ref 75. Copyright 2015 American Chemical Society.

While in photoiniferter polymerization, there is no photocatalyst that could reduce triplet oxygen, Qiao and co-workers showed that in the presence of an electron donor, trithiocarbonates can directly convert oxygen into inert superoxide by facilitating electron transfer from sacrificial tertiary amine to oxygen.^173^ While the oxygen was not entirely removed after 3 h of blue light irradiation (4 mW/cm^2^), oxygen levels were reduced from 21 to 1.9 vol%, enabling controlled polymerization. Importantly, this system does not rely on DMSO as a singlet oxygen scavenger and has been adapted to various solvents.^131^ Similarly as in oxygen-tolerant tolerant PET-RAFT, an extended induction period was observed. Further, the polymerization rate was reduced compared to that of the degassed reaction, and the apparent polymerization rate was dependent on the amine concentration. For xanthates, which undergo significantly faster photolysis than trithiocarbonates, polymerization through oxygen in which the RAFT agent consumes oxygen was reported.^174^ While this method does lead to partial RAFT agent deactivation and an increase in experimental molecular weight, good control was observed. This methodology was also successfully adapted to 3D printing.^100^ Oxygen tolerance is also observed in trithiocarbonate-mediated photoiniferter polymerization doped with xanthate, where it enables polymerization open to air in a 96 well-plate format, even though the CTA is consumed slowly under the presence of oxygen and irradiation.^164^

The alternative physical methods involve minimizing the headspace or filling the headspace with oil or inert immiscible solvent, which allows for enough oxygen to be removed so that polymerization can proceed in a controlled manner without prior deoxygenation, achieving high monomer conversions and good control, as demonstrated by the synthesis of multiblocks.^175^

## 10 Reactor Setup

One of the factors hampering our understanding and development of photopolymerization is a lack of standardized protocols. With manyreactor designs available, it is inevitable that different research groups develop their own platforms or reach for one of many commercial photoreactors. Due to the need for deep penetration of light into reactors, scale up system tends to favor flow reactors, where the tubular structure of the reactor ensures homogeneous and complete penetration of light. However, while commercial plug-and-play reactors can provide easy entry into the realm of flow chemistry, they are typically associated with high cost. Further, each commercial system comes with its own limitations, and the reproducibility of reaction conditions is obstructed by intellectual property, which often blocks the disclosure of crucial information, such as the exact makeup of tubing used for all kinds of phototransformations.^176^ While using the same setup by all researchers would increase uniformity, it would most definitely slow down the development of the process.

Independent of design, three key parameters to consider (and report) for the design of a given photoreactor are the wavelength of light, irradiance, (i.e., the amount of light that reaches the reaction vessel as power per area, typically reported in mW/cm^2^) and temperature. Additionally, the two latter parameters depend substantially on reactor geometry and material as well as reaction scale. Active mixing is also crucial due to both mass transfer and light penetration limitations. This section highlights important aspects of photoreactor design that are necessary to ensure research is reproducible and that the potential of photopolymerizations is fully utilized. For detailed examples of photoreactor setups, the reader is directed elsewhere.^36,42,177–183^

### 10.1 Light Source–Wavelength of Light and Photon Flux

The choice of the light source dictates the wavelength(s) of light and its intensity. Light sources can be classified into three categories based on how broad their emission spectrum is polychromatic, monochromatic, and quasi-monochromatic light sources (Figure 16).Halogen lamps, compact fluorescent lamps, and medium- and high-pressure mercury lamps fit into the first category. Light emitted by these sources encompasses a broad range of wavelengths. Long-pass filters eliminate shorter and transmit longer wavelengths, and band-pass filters that transmit only specific wavelength bands are commonly used with mercury lamps to make them more selective. High power lasers provide monochromatic emission; however, the cost and knowledge required for their setup and maintenance hinder their widespread use. LEDs (light-emitting diodes) as quasi-monochromatic light sources amalgamate narrow emission spectra with low cost. LEDs are available in a wide range of colors, including UV wavelengths, providing good alternatives to black light UV lamps, nail curing lamps, and laboratory thin-layer chromatography lamps.

**Figure 16:**
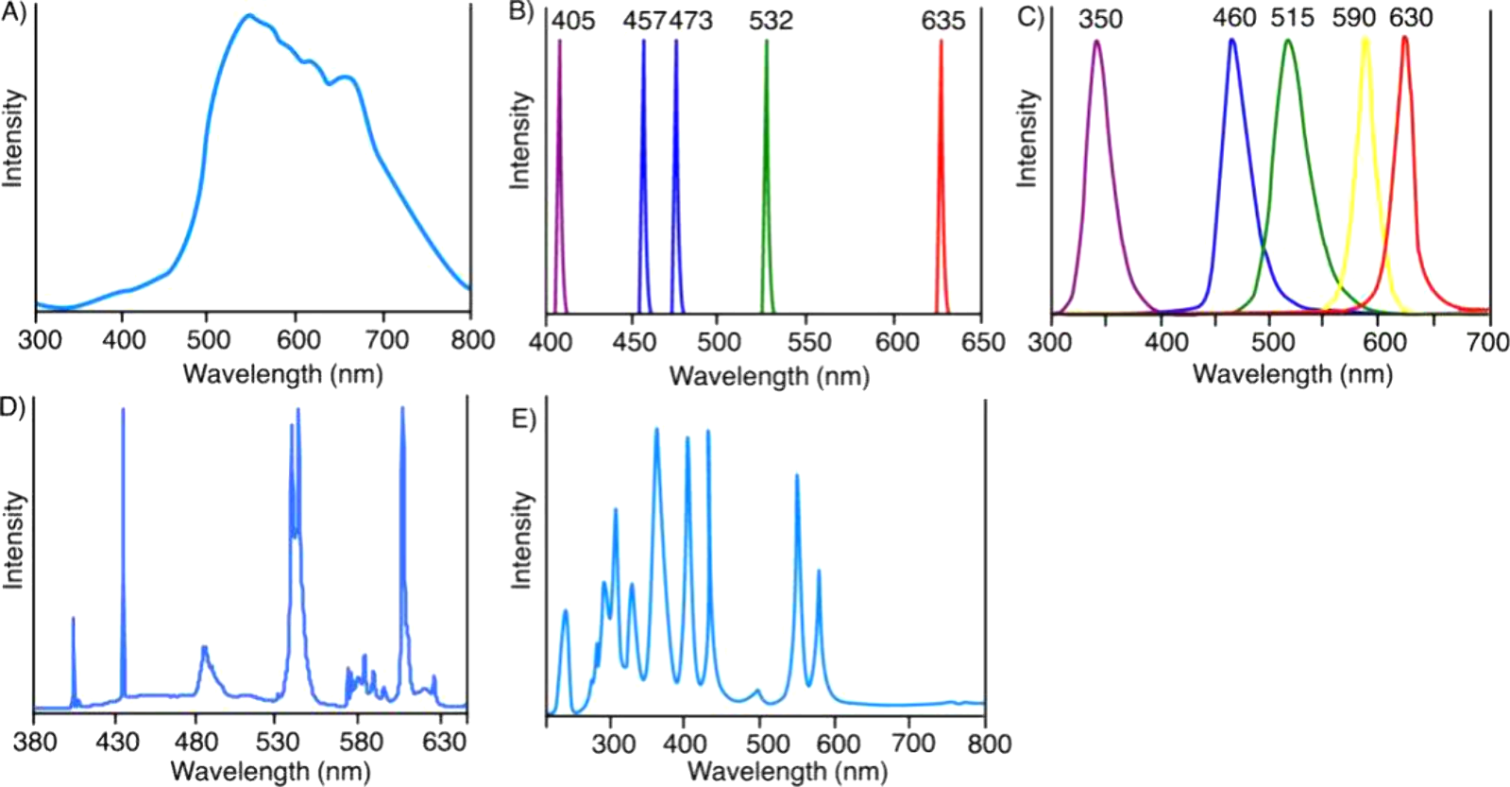
Typical emission spectra of (A) halogen lamps, (B) laser diodes at 405 (purple), 457 (blue), 473 (blue), 532 (green), and 635 nm (red), (C) UV lamp centered at 350 nm (left) and LEDs centered at 460 (blue), 515 (green), 590 (yellow), and 630 nm (red), (D) household compact fluorescent lamp, and (E) high-pressure mercury plasma arc-discharge lamp. Reproduced from Xiao, P.; Zhang, J.; Dumur, F.; Tehfe, M. A.; Morlet-Savary, F.; Graff, B.; Gigmes, D.; Fouassier, J. P.; Lalevee, J. Visible Light Sensitive Photoinitiating Systems: Recent Progress in Cationic and Radical Photopolymerization Reactions under Soft Conditions. *Prog.* *Polym. Sci.* **2015**, *41*, 3266. Copyright 2015 with permission from Elsevier. Reprinted from ref 22. Copyright 2016 American Chemical Society.

For low light intensity applications, LED strips with fixed single color or color changing capability are employed. Their intensity can be modulated either by altering the supplied current or changing the number of LEDs. Alternatively, surface-mounted high-lumen-output LEDs can be used. Due to their higher intensity, they usually require cooling to prevent LED damage, so they are typically mounted on heat sinks. Similarly, chip-on-board LEDs are usually made of multiple LED chips bonded directly to a substrate to form a single high-power module. Commercial high-power LED lamps or spotlights such as Kessil or EvoluChem are also available in a wide range of wavelengths.LED arrays are typically used for high throughput reaction screening in multiwell plates, which can be custom-built^42^ or sourced from commercial suppliers such as Amuza Inc. LEDA-x or Analytical Sales Lumidox.

To be able to fully control polymerization conditions, light intensity needs to be tunable, which can be typically exercised by employing a variable power supply or with a commercially available controller for specific lights such as Lumidox arrays or Kessil lamps; knowing the light intensity and emission wavelength is important for whatever light source is used. While the manufacturer often supplies emission spectra and do they not change over the lifespan of the light source (although they are affected to a degree by temperature), the light intensity depends on the exact reactor setup and will be affected by the material surrounding the light source and its distance from the reaction vessel as well as the beam angle, which describes how focused or dispersed light is. Light intensity will also decrease over time as the lamp deteriorates, so it is crucial to measure it regularly to ensure reproducibility.^184^ Emission spectra can be measured with a fluorimeter or spectroradiometer such as Solar Simulator Spectroradiometer from PV Measurements, while light intensity can be measured using a power meter, commonly used brands include Thorlabs and Newport. Light intensity should be normalized to area, and irradiance values received by the reactor should be reported, typically in mW/cm^2^.The wattage of the light source provides no information on the optical output, nor does the percentage intensity of the light source, as this is a relative and not an absolute value. Even irradiance measurement does not give a complete picture as the measurements are one-directional and will not discriminate different sizes and geometries of reaction vessels or take into account absorbance of the reaction vessel. Similarly, irradiance values are incomparable for samples irradiated from different directions (for example, from below vs in all directions). Ideally, photon flux defined as the number of photons per second per unit area (mol/m^2^/s, oftentimes reported in Einstein/m^2^/s) determined by actinometry or similar methods should be reported, giving information on the number of photons in spatiotemporal dimensions.^179,185,186^ However, such an approach is impractical due to time-consuming measurements that need to be performed for each reaction vessel and setup. A reproducible protocol should contain the following information: spectral characterization of the light source, irradiance value, geometric arrangement of the light source(s) and the photoreactor, positioning, dimensions, and material of the reaction vessel For experiments comparing different wavelengths of light, ideally the photon flux, not irradiance, should be matched between the experiments to maintain constant number of photons and only look at the difference arising from varying photon energy, not count.

### 10.2 Temperature Control

Classically, photochemistry is performed at room temperature as photons, not thermal energy, activate the photochemical process. Nonetheless, many reactions, particularly polymerizations, benefit from increased temperature as the photochemical pathway is only a part of the multistep mechanism (initiation, propagation, degenerative chain transfer, termination, etc.). On the other hand, some reactions, including ultrafast polymerizations, may require temperature below room temperature to maintain control and selectivity. It is, therefore, important to control, vary, and maintain the reaction temperature within a photoreactor. Further, higher-intensity LEDs, as well as mercury lamps, may require cooling to prevent heat damage. For small temperature changes, air is arguably the best heating/cooling medium, as it is fully transparent to light. Efficient air circulation also ensures a minimal temperature gradient across the reactor. However, for more significant heating or cooling applications the reactor can be submerged in water or a transparent oil or, in the case of a flow reactor, wrapped around a cylindrical heating–cooling block with a light source on the outside. Liquid circulating units can be used to control the reactor and light temperature and can be custom-built or purchased from commercial suppliers. An impressive example of temperature control was achieved in a 3D-printed open-access photoreactor using a combination of fans, heat sinks, and thermoelectric cooling elements, giving access to reaction temperatures ranging from −20 to 80 °C.^178^ Regardless of the photoreactor setup, the internal reaction temperature should be reported rather than the ambient reactor temperature.

### 10.3 Reactor Geometry—Batch and Flow Reactors

As already mentioned, reactor geometry and scale will have a substantial effect on light penetration and, as such, kinetics and control over photopolymerization. Typically, cylindrical and flat light sources are used, and both can be applied to batch and flow reactors (Figure 17). In a cylindrical setup, the light source is wrapped (LED strip) or positioned (multiple lamps) around the reaction vessel, giving 360° irradiation. If multiple vertical/spotlight lamps are used, it is essential to consider the focus of the light beam to avoid beam overlap and light hotspots. This setup can also be extended to flow reactors, where the tubing is wrapped around a framework and placed in the middle of the cylindrical photoreactor. Alternatively, the tubing framework can be placed outside the light source in a flow setup. In such a case, the framework needs to be transparent to the incoming light. While the tubing can also be wrapped directly around a light source without any framework, this can lead to poor temperature control. If the tubing is in close contact with light, the heat cannot efficiently dissipate. Flat light sources such as LED arrays are best suited for multiwell small-scale batch reactions and flow reactors, particularly microreactor chips; however, when placed vertically rather than horizontally, they can also provide lateral irradiation for cylinder-shaped batch reactors such as small Pyrex test tubes.

**Figure 17:**
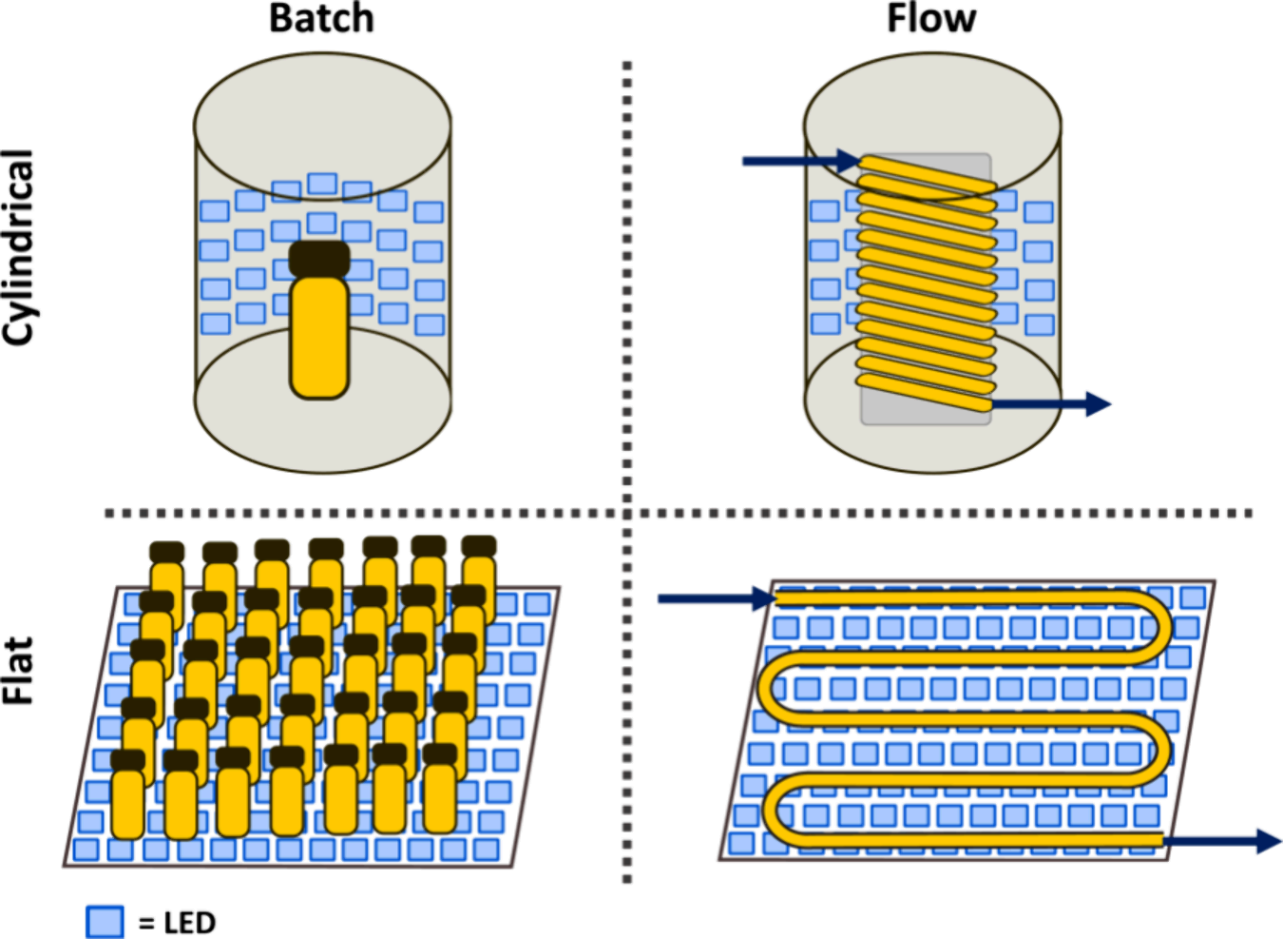
Typical geometries of photoreactors: cylindrical and one light source for batch and flow reactors.

Photopolymerizations in batch are simple to set up; however, they can suffer from limited light penetration. According to the Beer–Lambert law, the light absorbance is directly proportional to the concentration of the light-absorbing species (*c*) and the length of the light path (*l*): *A* = ε*cl*, where ε is the molar extinction coefficient. This severely impacts the light attenuation through the reaction medium, especially at high concentrations of photoactive species such as initiator or catalyst. Light gets absorbed very efficiently near the surface, and barely any light is transmitted to the inside of the reaction vessel as illustrated by the % transmittance (%T) of a PET-RAFT catalyst (Ru­(bpy)_3_Cl_2_) as a function of the path length (Figure 18).^187^ In a solution of 2.5 mM of this catalyst, more than 99.9% of incident light is absorbed within 0.1 cm path length. Even after 10-fold reduction in the catalyst concentration, only about 1% of light is transmitted past 1 cm path length. Although lower catalyst concentrations are typically used in polymerization, the issue of optical density will inevitably limit large-scale reactions. While reducing catalyst concentration would increase light penetration, this would slow down the polymerization as a catalyst is required for radical generation. Poor light transmittance can be circumvented by using flow chemistry, as the reaction mixture is constrained to narrow channels, ensuring good exposure to light. Furthermore, continuous flow approaches significantly expand available reaction conditions such as temperatures above the boiling point of the solvent under reduced pressure. Such systems are also easier to heat up/cool down than photo batch reactors.^149,188^ However, the viscosity of the medium must be balanced for an effective flow system.

**Figure 18:**
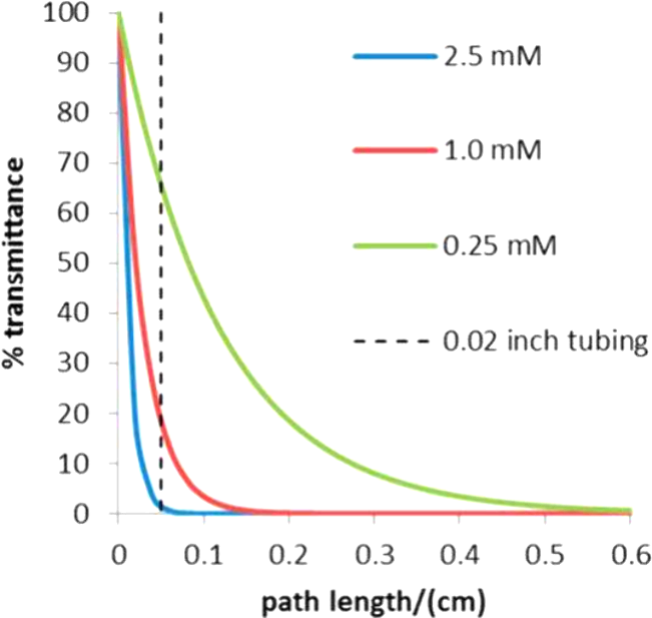
Percent transmittance plotted against the path length for different concentrations of [Ru­(bpy)_3_]­Cl_2_ in methanol. Reprinted from ref 187. Copyright 2017 American Chemical Society.

Within the realm of photopolymer chemistry, multiple polymerization techniques have been adapted to flow chemistry, such as ATRP,^111,189,190^ cobalt-mediated polymerization,^191,191^ and RAFT^172,192–194^ and reactor design and operational principles of continuous flow (micro)­reactors have been the subject of numerous reviews.^187,195,196^ Significant rate enhancements were reported for photoiniferter polymerization when translated to continuous flow.^69,149,152,197^ PET-RAFT has been successfully implemented in flow reactors without the need for deoxygenation, resulting in the fabrication of polymers with complex architectures, such as block copolymers with controlled dispersity^169,198,199^ and the production of antimicrobial polymers.^43^ Furthermore, PET-RAFT and flow reactors have been employed to screen a variety of experimental conditions. While increased radical concentration can lead to increased termination, polymers with narrow molecular weight distributions are generally achieved in continuous flow, which aligns with batch results. As flow reactors are characterized by increased light penetrability, care must be taken to avoid oversaturation of the system and irradiation past reaching full/desired monomer conversion. For this, careful kinetic analysis of the system is necessary; however, this is becoming significantly simplified for flow systems with the advancement of real-time reaction monitoring (inline and online) with SEC, NMR spectroscopy, and beyond.^200–203^

As viscous polymers move through narrow channels of flow reactors, deviation in fluid dynamics can be expected, which results in a residence time distribution as molecules travel through the reactor with different velocities and, hence, experience different reaction times. This can result in increased dispersities, particularly for viscous and heterogeneous systems such as emulsion polymerization.^204^ The severity of residence time distribution depends on several factors, including reactor design (tube diameter, coiling, etc.) and reaction kinetics—longer residence time usually results in poorer outcomes.^187^ If residence time distribution becomes a problem, it can be minimized by introducing droplet or slug flow, where slugs of the reaction mixture are compartmentalized within an immiscible mobile phase (liquid or gas). This approach has been shown to significantly improve the outcome of polymerizations in continuous flow.^172,205^ For a more detailed discussion on flow reactors, the reader is directed elsewhere.^187,206–208^

### 10.4 Reactor Material

Another important parameter to consider when designing a photoreactor is the material of the reactor housing and the reaction vial/tubing. The housing of the reactor can form a reflective environment to maximize photon capture by use of reflectors such as high-reflectance PTFE sheets,^180^ anodized aluminum reflectors^209^ or mirrors.^182^ For batch reactors, reaction vial material rarely constitutes a problem as typical laboratory glassware is made of borosilicate glass, which has a 275 nm wavelength cutoff and is suitable for most UV and all visible light reactions.^188^ For higher energy UV light, quartz would be a more appropriate material, with a cutoff around 170 nm.

The choice of material is more complicated for flow reactors. While borosilicate glass and quartz have substantially better light transparency and oxygen barrier properties to any polymeric tubing, glass tubular reactors have significant limitations. The reactors are very fragile and cannot be coiled. Further, as polymeric fittings are more difficult to fit on the ends of a glass flow cell, points of connection of the reactor with the reagent delivery system and analytical instruments can be potential points of oxygen ingress. Thermal radical polymerizations are typically run in stainless-steel tubing due to superior performance compared to polymeric tubing, but this is not possible for photoreactions.^210^ Alternatively, there is a wide range of polymeric tubings available to choose from. There is usually a trade-off between tubing transparency and oxygen permeability. Fluorinated polymers such as PTFE (polytetrafluoroethylene) and PFA (perfluoralkoxy) are characterized by high light transparency (wavelength cutoff at 50% transmittance 180–200 nm^211^), excellent chemical stability as well as good resistance to fouling^192^ but have higher oxygen permeability than ETFE (ethylene tetrafluoroethylene) and ECTFE (ethylene-chlorotrifluoroethylene) tubing. ETFE and ECTFE, on the other hand, are less transparent. Melker et al. compared tubing with different oxygen barrier properties for light-mediated photoredox polymerization of MMA with an iridium-based catalyst. The authors found that Halar tubing (ECTFE) outperformed PFA tubing, but the difference was marginal, especially considering a 35-fold difference in oxygen permeability between PFA and ECTFE. Halar tubing led to 15% conversion at 55 min while PFA only 11%.^212^ However, because of changes in oxygen permeability and light transparency, it is difficult to systematically assess the effect of oxygen ingress. Indeed, many successful photopolymerizations in PFA reactors were reported.^152,213,214^ Importantly, characteristics of the tubing will vary depending on the supplier, as the purity of tubing will affect both its barrier and transparency properties, and the presence of plasticizers can also affect polymerization results.

## 11 Bringing It All Together

Throughout this review, we discussed a variety of factors that affect the control and kinetics of photopolymerizations mediated with thiocarbonylthio compounds. Both photoiniferter and PET-RAFT polymerization require careful optimization to truly harness their potential. While more research is necessary to systematically study different polymerization conditions, further improve end-group fidelity, and reduce polymerization time, several well-studied factors affect the outcome of these light-based methods.

The wavelength of light depends on the choice of monomer and RAFT agent (photoiniferter polymerization) or photocatalyst (PET-RAFT). For both polymerization mechanisms, lower energy light is more selective. For PET-RAFT, longer wavelengths of light, such as green, red, or NIR minimize interference of the photoiniferter activation pathway with the PET mechanism.^72^ For photoiniferter polymerization, targeting the n−π* transition of the RAFT agent leads to faster and more controlled polymerization.^113^ For xanthate-mediated polymerization, one should opt for UV or purple light, while blue or green light should be preferred for trithiocarbonate- and dithiobenzoate-mediated polymerizations. Utilizing visible light for trithiocarbonates reduces the photodegradation of the RAFT agent and polymer end group.

To optimize kinetics and control over polymerization, acrylates and acrylamides are best paired with a trithiocarbonate with a secondary R group (PET-RAFT and photoiniferter polymerization), methacrylates with dithiobenzoate (PET-RAFT) or trithiocarbonate (PET-RAFT and photoiniferter polymerization) with a tertiary cyano substituted R group, and xanthates are best suited for less activated monomers (Figure 19, Table 1). As methacrylic monomers give rise to a relatively stable propagating radical, they are at an increased risk of photodegradation of the thiocarbonylthio end group. Conditions favoring propagation, such as high temperature and monomer concentration, as well as stopping the reaction at a lower monomer conversion, can lead to improved outcomes. The addition of tertiary amine improves both kinetics^124^ and control^123^ over the polymerization of methyl methacrylate. While increased light intensity can increase the polymerization rate, it can also lead to diminished control due to increased photodegradation. Alternatively, the polymerization rate can be tuned with temperature or reactant concentration, and in the case of PET-RAFT, the efficiency and loading of the catalyst (Table 1). Acrylates and acrylamides polymerize faster than methacrylates, so reaction time needs to be tuned accordingly. In PET-RAFT, the rate of polymerization also depends on the activity of the catalyst, which affects reaction time (Table 1).

**Figure 19:**
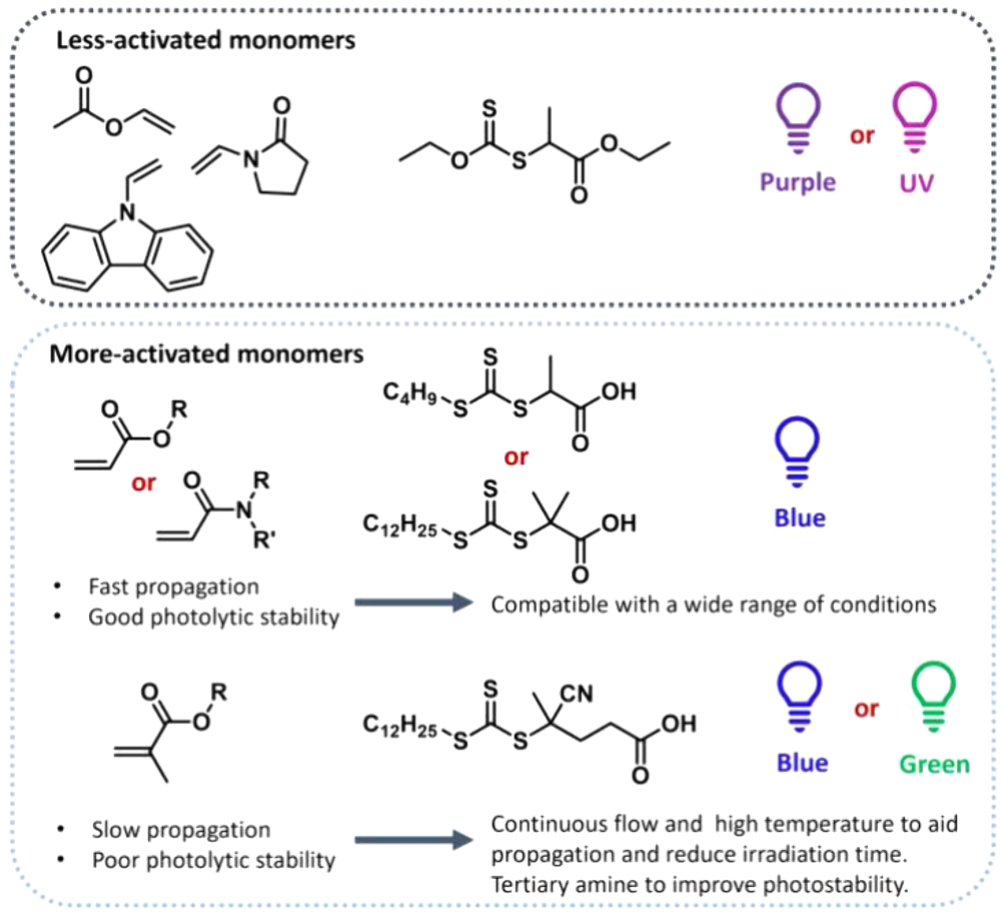
Summary of photoiniferter conditions for the polymerization of different monomers.

**Table 1:**
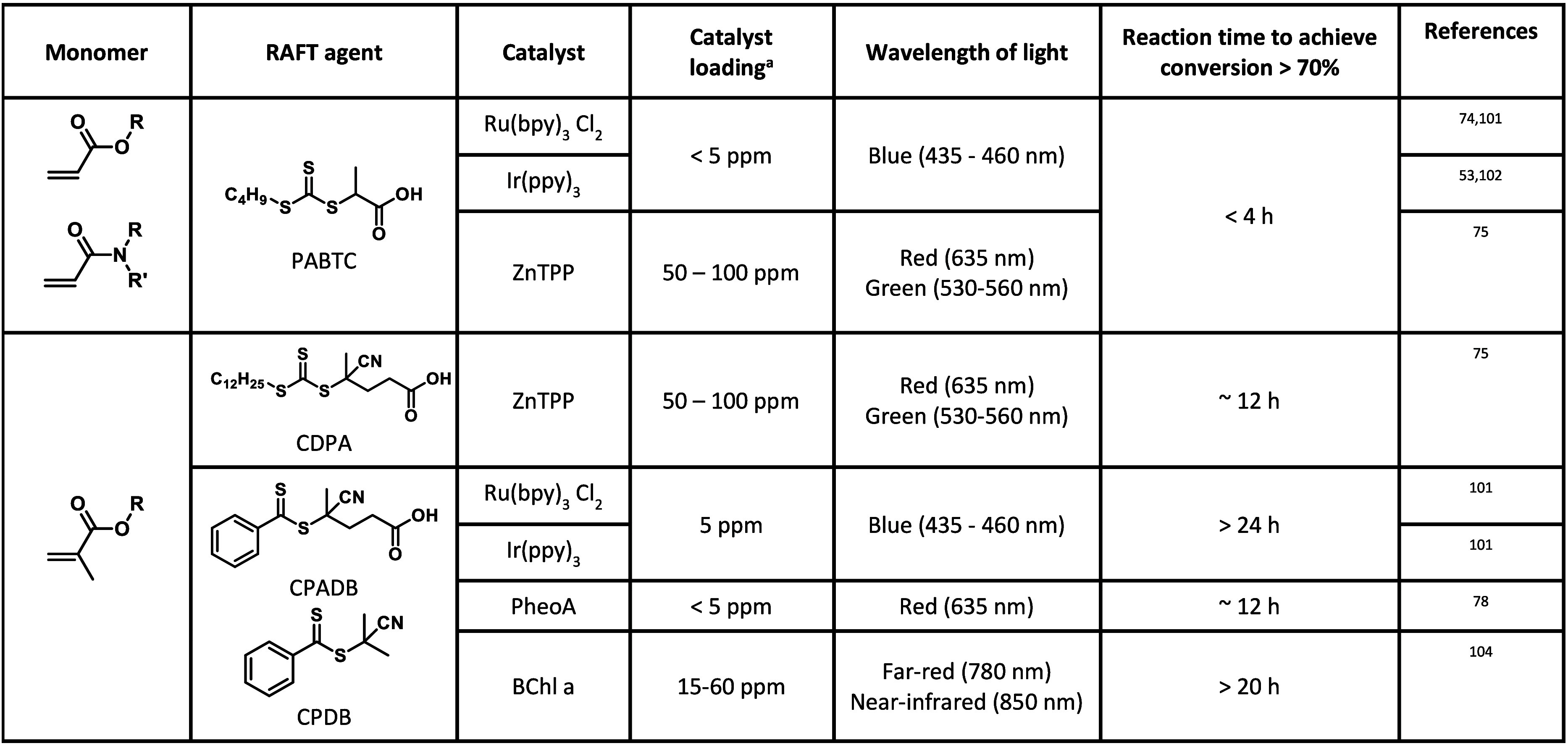
Summary of PET-RAFT Polymerization Conditions for Achieving High Monomer Conversion while Maintaining Good Control over Polymerization for Different More-Activated Monomers

## 12 Outlook

In comparison to conventional RAFT polymerization, photopolymerizations mediated with thiocarbonylthio compounds such as photoiniferter and PET-RAFT polymerizations offer several advantages, including precise spatial and temporal control over the polymerization process. Additionally, decoupling polymerization from thermal activation provides numerous benefits. Photopolymerizations also demonstrate superior potential in terms of monomer sequence control, maintaining living polymerization characteristics, and exhibiting higher tolerance to oxygen compared to thermal RAFT that relies on exogenous radical initiators. Over the past decade, the utility of visible light in photoiniferter and PET-RAFT polymerizations has become increasingly evident. While there are still numerous aspects that require further investigation, from a practical standpoint, photopolymerizations already enable processes that would be unachievable with conventional methods. Operational simplicity and tolerance toward a wide range of monomer functionalities further contribute to the popularity of such polymerization methods for the preparation of materials for a wide range of applications. In material design, photoiniferter and PET-RAFT polymerizations were exploited for applications such as 3D-printing^24,27,215,216^ and the facile modification of surfaces.^215,217–223^ Furthermore, photopolymerization facilitates the straightforward synthesis of complex macromolecules, such as multiblock copolymers, under ambient conditions.^66,67,160,167^ The number of applications of photoiniferter and PET-RAFT polymerizations is expected to expand as the understanding of the photochemical behavior of thiocarbonylthio compounds and mechanistic details of polymerizations are advanced. Finally, the integration of photopolymerizations mediated by thiocarbonylthio compounds with flow chemistry is gaining increasing attention. Flow chemistry is distinguished by its ability to operate under a broad spectrum of conditions, including high-pressure and high-temperature environments, which can further enhance the process control and reduce polymerization times. Additionally, flow reactors improve the efficiency of photochemical reactions by facilitating deeper light penetration compared to traditional batch reactors, a feature that is particularly beneficial when high concentrations of photoactive species are involved. Moreover, the integration of real-time monitoring with flow reactors enables dynamic kinetic analysis, offering valuable insights into photopolymerization mechanisms and opportunities for further process optimization.
